# Hypoxia‐Preconditioned BMSC‐Derived Exosomes Induce Mitophagy via the BNIP3–ANAX2 Axis to Alleviate Intervertebral Disc Degeneration

**DOI:** 10.1002/advs.202404275

**Published:** 2024-07-08

**Authors:** Yuxin Jin, Ouqiang Wu, Qizhu Chen, Linjie Chen, Zhiguang Zhang, Haijun Tian, Hao Zhou, Kai Zhang, Jianyuan Gao, Xinzhou Wang, Zhenyu Guo, Jing Sun, Kenny Yat Hong Kwan, Morgan Jones, Yan Michael Li, Ehsan Nazarzadeh Zare, Pooyan Makvandi, Xiangyang Wang, Shuying Shen, Aimin Wu

**Affiliations:** ^1^ Department of Orthopaedics Key Laboratory of Structural Malformations in Children of Zhejiang Province Key Laboratory of Orthopaedics of Zhejiang Province The Second Affiliated Hospital and Yuying Children's Hospital of Wenzhou Medical University Wenzhou Zhejiang 325000 China; ^2^ Department of Orthopaedic Surgery Shanghai Sixth People's Hospital Affiliated to Shanghai Jiao Tong University School of Medicine Shanghai 200025 China; ^3^ Shanghai Key Laboratory of Orthopedic Implants Department of Orthopedics Ninth People's Hospital Shanghai Jiao Tong University School of Medicine Shanghai 200011 China; ^4^ Department of Orthopaedics and Traumatology Li Ka Shing Faculty of Medicine The University of Hong Kong 5/F Professorial Block Queen Mary Hospital 102 Pokfulam Road Pokfulam Hong Kong SAR China; ^5^ Spine Unit The Royal Orthopaedic Hospital Bristol Road South Northfield Birmingham B31 2AP UK; ^6^ The minimaly invasive Brain and Spine Institute, Department of Neurosurgery State University of New York Upstate medical university 475 Irving Ave, #402 Syracuse NY 13210 USA; ^7^ School of Chemistry Damghan University Damghan 36716–45667 Iran; ^8^ University Centre for Research & Development Chandigarh University Mohali, Punjab 140413 India; ^9^ Department of Biomaterials, Saveetha Dental College and Hospitals, SIMATS Saveetha University Chennai 600077 India; ^10^ Department of Orthopaedics Key Laboratory of Musculoskeletal System Degeneration and Regeneration Translational Research of Zhejiang Province Sir Run Shaw Hospital Zhejiang University School of Medicine Hangzhou 310000 China

**Keywords:** bone marrow mesenchymal stem cells, exosomes, hypoxia‐preconditioned mesenchymal stem cells, Intervertebral disc degeneration, matrix reconstruction, mitophagy

## Abstract

Intervertebral disc degeneration (IVDD) is a chronic degenerative disease involving the aging and loss of proliferative capacity of nucleus pulposus cells (NPCs), processes heavily dependent on mitochondrial dynamics and autophagic flux. This study finds that the absence of BCL2/adenovirus E1B 19 kDa interacting protein 3 (BNIP3) is associated with senescence‐related NPC degeneration, disrupting mitochondrial quality control. Bone marrow mesenchymal stem cells (BMSCs) have multidirectional differentiation potential and produce extracellular vesicles containing cellular activators. Therefore, in this study, BMSCs are induced under hypoxic stimulation to deliver BNIP3‐rich extracellular vesicles to NPCs, thereby alleviating aging‐associated mitochondrial autophagic flux, promoting damaged mitochondrial clearance, and restoring mitochondrial quality control. Mechanistically, BNIP3 is shown to interact with the membrane‐bound protein annexin A2 (ANXA2), enabling the liberation of the transcription factor EB (TFEB) from the ANXA2‐TFEB complex, promoting TFEB nuclear translocation, and regulating autophagy and lysosomal gene activation. Furthermore, a rat model of IVDD is established and verified the in vivo efficacy of the exosomes in repairing disc injuries, delaying NPC aging, and promoting extracellular matrix (ECM) synthesis. In summary, hypoxia‐induced BMSC exosomes deliver BNIP3‐rich vesicles to alleviate disc degeneration by activating the mitochondrial BNIP3/ANXA2/TFEB axis, providing a new target for IVDD treatment.

## Graphical abstract

This study delves into the therapeutic efficacy of hypoxia‐induced bone marrow mesenchymal stem cell exosomes enriched with BCL2/adenovirus E1B 19 kDa interacting protein 3 (BNIP3) in mitigating intervertebral disc degeneration (IVDD). Through mechanistic insights, this study explores the restoration of mitochondrial quality control and delays in nucleus pulposus cell aging, offering promising avenues for IVDD treatment.

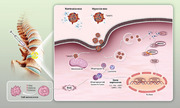

## 1 Introduction

Lower back pain is a prevalent global health issue that significantly impacts individuals worldwide, leading to substantial healthcare costs and disability.^[^ ^1^ ^]^ From the pathophysiological perspective, intervertebral disc degeneration (IVDD) is a significant factor contributing to lower back pain.^[^ ^2^ ^]^ Anatomically, the intervertebral disc is composed of a central nucleus pulposus (NP), an outer annulus fibrosus (AF), and upper and lower cartilaginous endplates (CEP).^[^ ^3^ ^]^ Disc degeneration is attributed to a multitude of factors, including diminished water content within the NP, reduced elasticity of the aging AF, and ossification of the CEP.^[^ ^4^ ^]^ Cellular senescence, inflammation, and damage to the mitochondria, essential organelles for cellular energy production and reactive oxygen species (ROS) generation, collectively contribute to reduced cell density and compromised extracellular matrix (ECM) degradation and synthesis in the NP.^[^ ^5^ ^]^ The current study aimed to investigate the pathological mechanisms underlying IVDD at the molecular level to identify novel targets for enhancing the treatment of this condition.

Bone marrow‐derived mesenchymal stem cells (BMSCs) possess remarkable multilineage differentiation and cellular regeneration modulation abilities, as well as low immunogenicity. These qualities have made BMSCs a highly sought‐after cellular system for treating orthopedic disorders.^[^ ^6^ ^]^ Typically, stem cells possess the capacity to migrate to damaged tissues and repair impaired cells through various mechanisms such as direct differentiation, cell–cell interactions, and paracrine release of active cytokines.^[^ ^7^ ^]^ Theoretically, stem cell transplantation holds promise as an excellent option for cell repair and regeneration. However, the intercellular microenvironment, ischemia, inflammation, oxidative stress, limited homing ability, lack of targeted migration, and low post‐transplantation survival rates present significant challenges for stem cell therapy.^[^ ^8^ ^]^ Therefore, researchers are currently focusing on exploratory studies of stem cells.

Extracellular vesicles, specifically exosomes, serve as vital modes of paracrine secretion from stem cells, enabling the delivery of parent cell characteristics and corresponding genes to recipient cells through noncellular contact mechanisms.^[^ ^9^ ^]^ Typically, in vitro experiments involve exposing BMSCs to normoxic conditions (21% O^2^), which significantly deviate from the low‐oxygen microenvironment (2–8%) within the human body. Such culture conditions may be critical factors that influence the potential of mesenchymal stem cells.^[^ ^10^ ^]^ Hypoxia enhances the intrinsic activity of stem cells by reducing glucose consumption, lactate levels, and the release of cytochrome c. Additionally, extracellular vesicles derived from low‐oxygen‐treated cells exhibit promising therapeutic effects in various disease models.^[^ ^11^ ^]^ Therefore, we used hypoxic preconditioning to induce related changes in intracellular metabolism, enhancing the therapeutic efficacy of the resulting exosomes.^[^ ^12^ ^]^

**Scheme 1:**
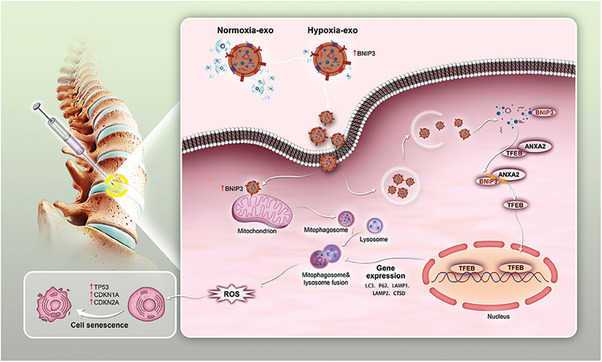
Revealing the exosome therapy strategy of hypoxia preconditioning and its specific molecular mechanisms in IVDD.

BCL2/adenovirus E1B 19 kDa interacting protein 3 (BNIP3) is a mitochondria‐targeted protein that shares homology with BCL2 interacting protein 3 like (BNIP3L) and potentially possesses a BH3 domain. It is localized to the outer mitochondrial membrane.^[^ ^13^ ^]^ The phosphorylation of serine residues in the LC3‐interacting region (LIR) of the N‐terminus of BNIP3 facilitates its interaction with LC3B.^[^ ^14^ ^]^ The C‐terminus of BNIP3 contains multiple phosphorylation sites, including TM, S/T, and RRLT sequences. Under oxidative stress or hypoxic conditions, complete phosphorylation of the BNIP3 C‐terminus attenuates its cytotoxic effects.^[^ ^15^ ^]^ BNIP3 functions as a mitochondrial autophagy receptor activated under hypoxia and nutrient deprivation to maintain mitochondrial quality control and safeguard cell survival.^[^ ^16^ ^]^ Studies have demonstrated that BNIP3 plays a role in regulating mitochondrial autophagic flux and mitochondrial–lysosomal crosstalk.^[^ ^4^, ^17^ ^]^ However, the exact role and specific molecular mechanisms by which BNIP3 maintains intervertebral disc integrity and mitochondrial quality control in nucleus pulposus cells (NPCs) remain unclear. Therefore, in this study, we examined the regulatory network of BNIP3 in mitochondrial autophagy, which coordinates mitochondrial quality control and promotes the formation of autolysosomes to eliminate damaged mitochondria, thereby delaying senescence in NPCs (**Scheme** **1**).

## 2 Results

### 2.1 Differential Expression of BNIP3 and Assessment of Mitochondrial Damage in Degenerated NP Tissue

**Figure 1:**
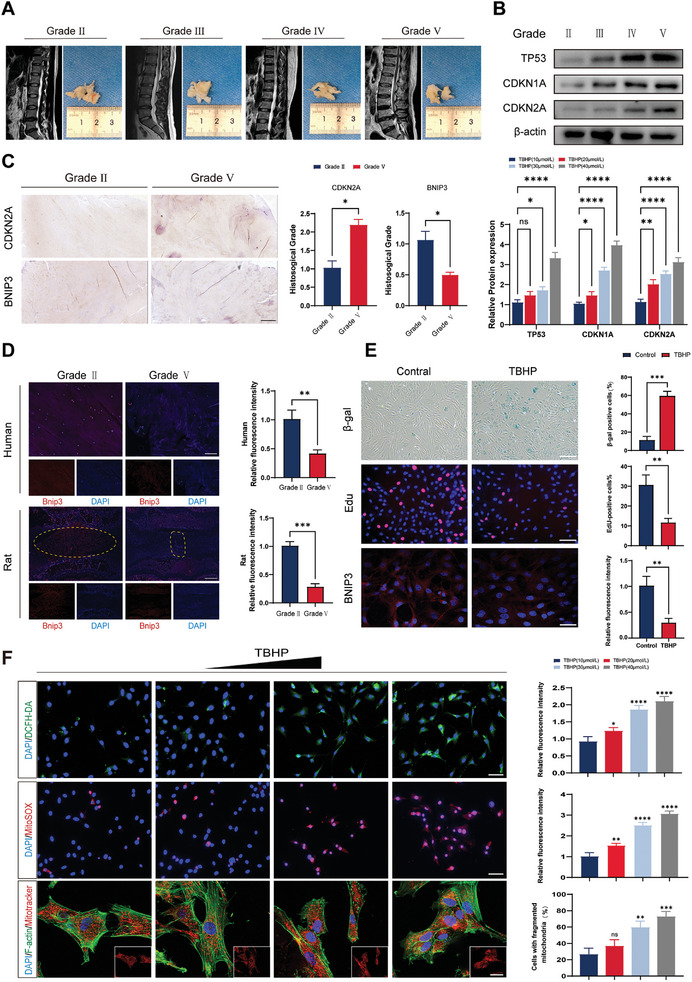
Mitochondrial damage and BNIP3 downregulation in senescent degenerating nucleus pulposus tissues. A) T2‐weighted magnetic resonance imaging (MRI) was utilized to procure human nucleus pulposus tissues exhibiting varying degrees of degeneration, as per the Pfirrmann grading system. B) Senescence‐associated proteins (tumor protein 53 [TP53], cyclin‐dependent kinase inhibitor 1A [CDKN1A], and cyclin‐dependent kinase inhibitor 2A [CDKN2A]) in degenerated tissues were quantitatively evaluated via western blotting. *n* = 3, *p < 0.05; **p < 0.01; ***p < 0.001; ****p < 0.0001; ns, not significant. C) Immunohistochemical staining was performed to examine the expression of CDKN2A and BNIP3 in different degenerating tissues. *n* = 3, scale bar: 500 µm. *p < 0.05. D) Tissue fluorescence assessment of BNIP3 expression in degenerating human and rat nucleus pulposus tissue. Human tissue: *n* = 3, scale bar: 100 µm. Rat tissue: *n* = 3, scale bar: 200 µm. **p < 0.01; ***p < 0.001. E) Nucleus pulposus cells were stimulated with tert‐butyl peroxide (TBHP), and differences between the TBHP‐treated and control groups were assessed by β‐galactosidase, EdU, and immunofluorescence staining. β‐gal and EdU: *n* = 3, scale bar: 100 µm. Immunofluorescence staining for BNIP3; scale bar: 50 µm. **p < 0.01; ***p < 0.001. F) Mitochondrial function in isolated human nucleus pulposus cells was evaluated by 2′−7′ dichlorofluorescein (DCFH‐DA), MitoSox, and MitoTracker assays following exposure to TBHP at concentrations of 10, 20, 30, and 40 µmol/mL. DCFH‐DA: *n* = 3, scale bar: 100 µm. MitoSox: *n* = 3, scale bar: 100 µm. MitoTracker: *n* = 3, scale bar: 20 µm. *p < 0.05; **p < 0.01; ***p < 0.001; ****p < 0.0001; ns, not significant. Data are expressed as mean ± standard deviation.

The NP is centrally located within intervertebral disc tissue, and any damage to NPCs inevitably contributes to the development of IVDD. In addition, age‐associated mitochondrial dysfunction in NP tissues has been identified as a key factor in the irreversible degeneration caused by IVDD. In this study, we assessed the severity of IVDD using the Pfirrmann grading system based on magnetic resonance imaging (MRI; Table S1, Supporting Information). We collected 16 NP samples from different patients with IVDD, categorized them based on the degree of degeneration observed in imaging (**Figure** **1**A), and examined the protein expression of the senescence‐associated factors tumor protein 53 (TP53)/p53, cyclin‐dependent kinase inhibitor 1A (CDKN1A)/p21, and cyclin‐dependent kinase inhibitor 2A (CDKN2A)/p16 within the samples (Figure 1B). Our findings revealed elevated expression levels of TP53/p53, CDKN1A/p21, and CDKN2A/p16 in the severe IVDD group (Grade III/IV) compared to those in the mild IVDD group (Grade II/III). To investigate BNIP3 expression in degenerating NP tissues, we performed immunohistochemical staining for CDKN2A and BNIP3. Our results revealed decreased expression of BNIP3 and upregulation of CDKN2A in the grade V group compared to levels in the grade II group. (Figure 1C). Notably, immunofluorescence analysis of rat IVDD and degenerating human NP tissues also indicated downregulation of BNIP3 (Figure 1D) in both models. To simulate an in vitro model of IVDD, we treated NPCs with tert‐butyl peroxide (TBHP) (Figure 1E). This treatment resulted in an increased number of β‐galactosidase‐positive cells and reduced the proliferative efficiency of EdU‐labeled cells, suggesting that NPCs are prone to senescence upon exposure to TBHP. Similarly, after TBHP treatment of NPCs, we observed a significant accumulation of ROS in cells upon staining with 2′−7′ dichlorofluorescein (DCFH‐DA). Furthermore, the mitochondrial superoxide indicator MitoSox demonstrated an accumulation of ROS within the mitochondria. Concurrently, MitoTracker staining revealed a shift in mitochondrial morphology from elongated strips to fragmented, dot‐like structures or a non‐fluorescent appearance (Figure 1F). These results suggest that disrupted mitochondrial homeostasis in senescent NPCs contributes to the persistent and irreversible degeneration of these cells.

In this regard, we concluded that differences existed between the degenerated NP tissue and undegenerated groups, as evidenced by the upregulation of senescence‐related genes, restriction of cell proliferation, and downregulation of the mitochondrial autophagy gene BNIP3. We successfully constructed a cellular model of senescence and mitochondrial damage in NPCs using TBHP, which lays the foundation for subsequent studies.

### 2.2 Characterization and Identification of Exosomes for Restoration of NPC Extracellular Matrix Homeostasis

**Figure 2:**
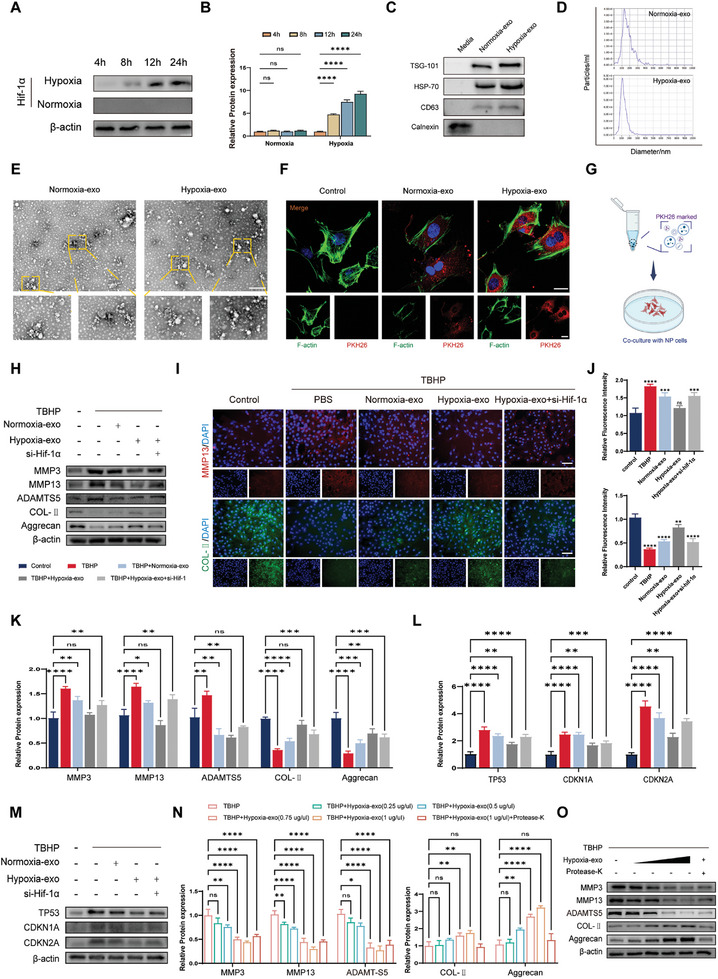
Characteristics and functional analysis of exosomes in human bone marrow‐derived mesenchymal stem cells (HBMSCs) subjected to hypoxic conditions. A) Hypoxia‐inducible factor 1 (HIF‐1) expression in HBMSCs was detected by western blotting after subjecting them to hypoxia for 4, 8, 12, or 24 h. B) The quantitative analysis of western blotting: *n* = 3, ****p < 0.0001; ns, not significant. C) Surface marker proteins (tumor susceptibility 101 [TSG101], heat shock protein 70 [HSP70], CD63 and endoplasmic reticulum protein [Calnexin]) of exosomes in hypoxia‐treated cells (hypoxia‐exo) and those of the normoxia‐treated group (normoxia‐exo) were quantified using western blotting. D) Nanoparticle tracking analysis revealed that both hypoxia‐exo and normoxia‐exo had a size distribution ranging from 40–150 nm. E) Differences in exosome morphology were observed under transmission electron microscopy. Scale bar: 5 µm. F) Exosomes were labeled with PKH26 and co‐incubated with nucleus pulposus cells for 24 h. Laser confocal microscopy demonstrated that hypoxia‐exo had stronger endocytosis efficiency than did normoxia‐exo. Scale bar: 20 µm. G) A schematic diagram was created to illustrate the co‐incubation process. H) Western blot analysis of catabolism‐ (matrix metallopeptidase 3 [MMP3], matrix metallopeptidase 13 [MMP13], and ADAM metallopeptidase with thrombospondin type 1 motif 5 [ADAMTS5]) and anabolism‐related markers (collagen II [COL‐II] and aggrecan) after different treatments. I) Immunofluorescence staining of biomarkers related to matrix catabolism (MMP13; red) and anabolism (COL‐II; green). Scale bar: 100 µm. J) Quantitative analysis of MMP13 and COL‐II fluorescence intensity. *n* = 3, **p < 0.01; ***p < 0.001; ****p < 0.0001; ns, not significant. K) Quantitative analysis of MMP3, MMP13, ADAMTS5, COL‐II, and aggrecan expression. *n* = 3, *p < 0.05; **p < 0.01; ***p < 0.001; ****p < 0.0001; ns, not significant. L) Quantitative analysis of the senescence‐related markers tumor protein 53 (TP53)/p53, cyclin dependent kinase inhibitor 1A (CDKN1A)/p21, and cyclin dependent kinase inhibitor 2A (CDKN2A)/p16 expression. *n* = 3, **p < 0.01, ****p < 0.0001. M) Western blot analysis of senescence‐related markers (TP53/p53, CDKN1A/p21, and CDKN2A/p16). N) Quantitative analysis of exosome function after proteinase K pretreatment. *n* = 3, *p < 0.05; **p < 0.01; ***p < 0.001; ****p < 0.0001; ns, not significant. O) Western blot analysis of exosome function after proteinase K pretreatment. Data are expressed as mean ± standard deviation. BMSC, bone mesenchymal stem cell.

Hypoxia, a condition characterized by a limited oxygen supply, can activate cellular responses in the form of stress‐induced release or adaptive changes. These cellular adaptations to hypoxic environments may be beneficial.^[^ ^18^ ^]^ Therefore, we conducted experiments on human bone marrow‐derived mesenchymal stem cells (HBMSCs) to investigate the effects of hypoxic chambers on the production of hypoxia‐inducible factor 1 (HIF‐1). We subjected the HBMSCs to different durations of hypoxia (4, 8, 12, and 24 h) in chambers maintained at 37 °C under 5% CO_2_ and 2% O_2_. Our results showed that the intracellular production of HIF‐1 peaked after 24 h of hypoxia treatment (**Figure** **2**A,B). Western blot analysis of exosome surface markers (tumor susceptibility 101 [TSG101], heat shock protein 70 [HSP70], and CD63) revealed slightly elevated expression of exosomes in hypoxia‐treated cells (hypoxia‐exo) compared to that in the exosomes of the normoxia‐treated group (normoxia‐exo) (Figure 2C). Nanoparticle tracking analysis results demonstrated that both hypoxia‐exo and normoxia‐exo had particle diameters of ≈100 nm (Figure 2D,E). Hypoxia pretreatment weakly altered the exosome particle size, while the appearance, size, and basic characteristics of both exosome groups remained consistent. To further assess the endocytic effect of exosomes on NPCs, we labeled the exosomes with PKH26 and co‐incubated them with NPCs for 24 h (Figure 2F,G). Using high‐magnification fluorescence microscopy, we observed that the hypoxia‐exo group exhibited superior endocytosis compared with that of the normoxia‐exo group.

We then analyzed expression of catabolism (matrix metallopeptidase [MMP13]) and anabolism (collagen II [COL‐II]) markers in NPCs by western blotting, and after TBHP treatment, we exogenously added normoxia‐treated (normoxia‐exo) or hypoxia‐treated BMSC exosomes (hypoxia‐exo) to these cells while treating the control group with phosphate‐buffered saline (PBS) only (Figure 2H,K). The results showed that treatment with hypoxia‐exo was superior to that with normoxia‐exo in terms of ECM rescue. The western blotting and immunofluorescence results were consistent, and treatment with hypoxia‐exo downregulated the expression of the catabolism markers matrix metallopeptidase 3 (MMP3), MMP13, and ADAM metallopeptidase with thrombospondin type 1 motif 5 (ADAMTS5) and upregulated the expression of the anabolism markers COL‐II and aggrecan (Figure 2I,J). We further validated the efficacy of hypoxia‐exo at the mRNA level with quantitative PCR experiments; the results were congruent with the western blot findings (Figure S1, Supporting Information). To confirm the critical role of HIF‐1α in HBMSCs, we used siRNA targeting *HIF‐1α* to knock down its expression in BMSCs, which resulted in partial dysfunction of exosomal components. In the knockdown of HIF‐1α (si‐Hif‐1α) treatment group, we found that the improvements provided by treatment with hypoxia‐exo were partially reversed (Figure 2H–K). In addition, we evaluated the effects of hypoxia on senescence‐related markers in NPCs. Western blot analysis of TP53/p53, CDKN1A/p21, and CDKN2A/p16 showed that hypoxia‐exo treatment effectively reversed the TBHP‐induced senescence phenotype of the NPCs (Figure 2M,L). Similarly, in si‐Hif‐1α‐treated BMSCs, a partial loss of function in exosomes secreted under hypoxic conditions was observed (Figure 2M,L). In addition, the protective effect of hypoxia‐exo was partially attenuated by pretreatment with proteinase K, suggesting that the therapeutic effect of exosomes depends on intravesicular cargo delivery (Figure 2N,O).

### 2.3 Hypoxia‐Exo Ameliorates IVDD in Rats In Vivo

**Figure 3:**
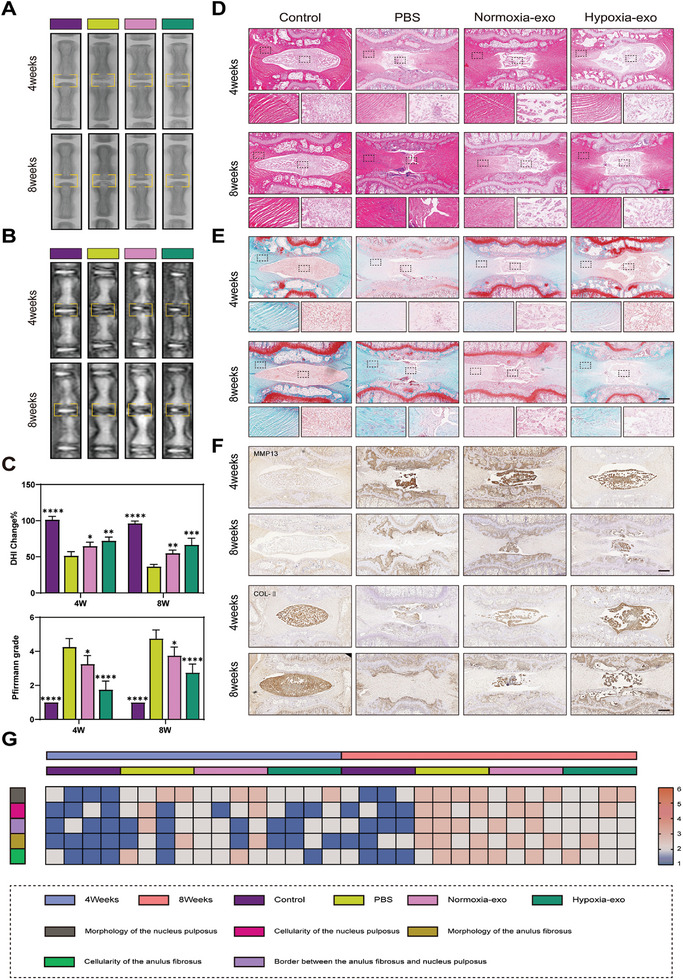
Imaging assessment and histologic evaluation after exosomal treatment in vivo. A) Postoperative X‐ray assessment of rats with intervertebral disc degeneration (IVDD) at 4 and 8 w (*n* = 4). B) Postoperative magnetic resonance imaging (MRI) assessment of rats at 4 and 8 w (*n* = 4). C) Quantitative analysis of rat disc height index scores and Pfirrmann grades. *p < 0.05; **p < 0.01; ***p < 0.001; ****p < 0.0001; ns, not significant. D) Hematoxylin and eosin (HE) staining images of rat caudal spines 4 and 8 w after surgery (*n* = 4). Scale bar: 1 mm. E) Safranin‐O/Fast Green (SF) staining images 4 and 8 w after surgery (*n* = 4). Scale bar: 1 mm. F) Immunohistochemistry of matrix metallopeptidase 13 (MMP13) and collagen II (COL‐II) 4 and 8 w after surgery (*n* = 4). Scale bar: 500 µm. G) Quantitative analysis of the data was plotted as heat maps based on the morphology of the nucleus pulposus, cellularity of the nucleus pulposus, border between the anulus fibrosus and nucleus pulposus, morphology of the anulus fibrosus, and cellularity of the anulus fibrosus. Data are expressed as mean ± standard deviation.

To investigate the influence of exosomes on IVDD in vivo, we established a rat model of IVDD and radiographically evaluated the rats according to different postoperative periods (four and eight weeks) to observe IVDD progression and changes in disc height index (DHI). After surgery, PBS, normoxia‐exo, or hypoxia‐exo was injected into the intervertebral discs of the rats. Using X‐ray imaging and MRI, we assessed the changes in the intensity of the DHI (Figure S2, Supporting Information) and the high signal intensity of the NP in the T2‐weighted phase (Table S1, Supporting Information). In the PBS‐injected group, the DHI was significantly decreased, characterized by cone collapse, intervertebral space wear, and osteophyte formation (**Figure** **3**A). This finding was confirmed by MRI (Figure 3B). While all experimental groups showed a loss of NP signal, this was most evident in the PBS‐injected group, followed by the normoxia‐exo‐treated group, whereas the hypoxia‐exo‐treated group showed the smallest change. The differences in the degree of degeneration between the groups were small at four weeks and significant at eight weeks (Figure 3C).

In addition to radiographic evidence, histology was used to evaluate the efficacy of the different treatments. Specifically, we utilized hematoxylin and eosin (HE) and Safranin‐O/Fast Green (SF) staining of rat disc sections (Table S2, Supporting Information). Four weeks after treatment with PBS, we observed compression‐induced deformation of the NP, leading to a reduction of ≈20–25% in its area. The normoxia‐exo‐treated group showed partial amelioration, whereas the hypoxia‐exo‐treated group demonstrated the most pronounced therapeutic effect. After eight weeks, the PBS‐treated group exhibited a nearly complete disappearance of NPCs and replacement of the intervertebral space with inwardly extending chondrocytes compared with features in the control group. In the normoxia‐exo‐treated group, IVDD progression was mitigated, and the hypoxia‐exo‐treated group displayed the most significant amelioration (Figure 3D). After SF staining, the medullary tissue appeared red owing to collagen enrichment, whereas the NP tissue appeared blue owing to fiber enrichment. In the PBS‐treated group, we observed the lowest ratio of medullary tissue area compared to that in the control group, and the deficient medullary tissue was replaced by a blue fibrous ring, features which were second‐most common in the normoxia‐exo‐treated group. In contrast, the red color, indicative of collagen enrichment, was still present in a larger area in the hypoxia‐exo‐treated group eight weeks after the operation, indicating superior treatment efficacy (Figure 3E).

MMP13 immunohistochemistry demonstrated the following (Figure 3F): in the control group, NPCs exhibited a lighter brown color; in the PBS‐treated group, the NP area decreased, and the intensity of histone staining increased compared to those in the control group; in the normoxia‐exo‐treated group, the degree of brown coloration was slightly decreased relative to that in the PBS‐treated group; and the hypoxia‐exo‐treated group exhibited a further decrease in brown coloration, indicating downregulation of MMP13.

The COL‐II immunohistochemistry results revealed that the control group exhibited the strongest brown coloration (Figure 3F). Conversely, the PBS‐treated group displayed the lightest brown coloration, with most of the NP retaining its original color. In the normoxia‐exo‐treated group, the staining intensity was slightly higher than that in the PBS‐treated group. Notably, the hypoxia‐exo‐treated group displayed a superior staining intensity compared to both the normoxia‐exo‐treated and PBS‐treated groups, confirming the efficacy of hypoxia‐pretreated BMSC exosomes both in vivo and in vitro in rats. The results of the HE and SF staining and immunohistochemistry scoring are presented as heat maps in Figure 3G.

### 2.4 Genetic Differences in Hypoxia‐Preconditioned Exosomes

**Figure 4:**
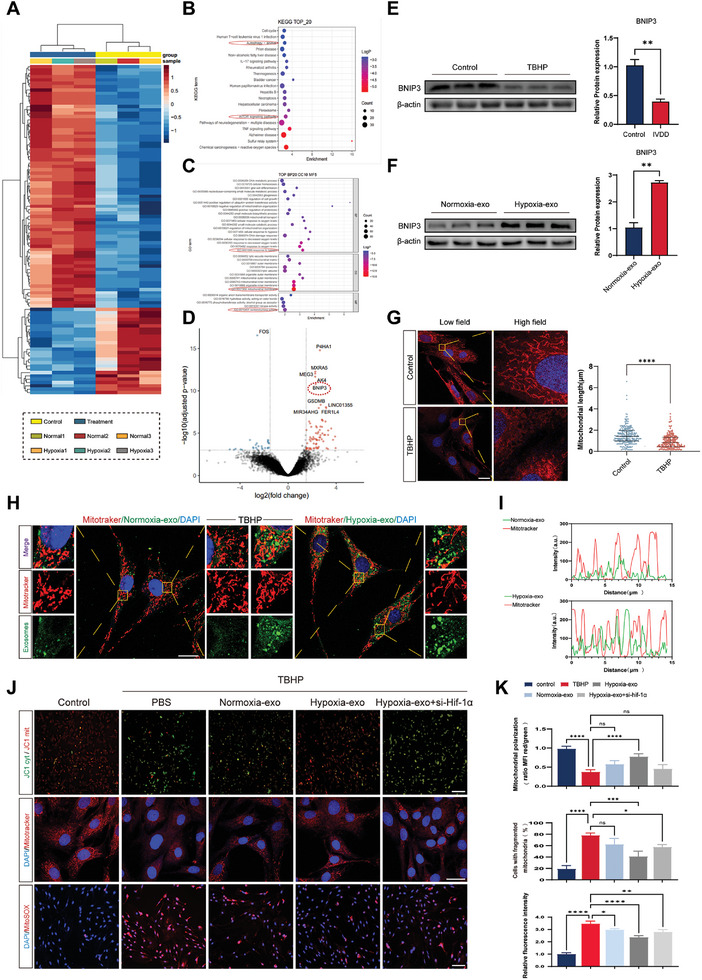
Hypoxic exosomes protect nucleus pulposus cells (NPCs) from oxidative stress‐induced mitochondrial dysfunction. A) Differential gene enrichment maps in hypoxic and normoxic exosomes. B) Differential gene pathway enrichment analysis (Kyoto Encyclopedia of Genes and Genomes [KEGG] analysis). C) Gene ontology (GO) analysis of differential genes. D) Volcano plot showing differential gene expression in hypoxic and normoxic groups. False discovery rate < 0.001, logFC > 1.5. E) Western blot analysis of BCL2/adenovirus E1B 19 kDa interacting protein 3 (BNIP3) levels in tert‐butyl peroxide (TBHP)‐treated NPCs. *n* = 3, **p < 0.01. F) Western blot analysis of BNIP3 levels in NPCs treated with hypoxic versus normoxic exosomes. *n* = 3, **p < 0.01. G) Mitochondrial length measurements of NPCs under TBHP stimulation. *n* = 3, scale bar: 20 µm. ****p < 0.0001. H,I) Co‐localization of PKH67‐labeled exosomes with MitoTracker‐labeled mitochondria. *n* = 3, scale bar: 20 µm. J) Fluorescence analysis of hypoxic and normoxic exosomes for mitochondrial membrane potential (JC‐1; scale bar: 100 µm) and mitochondrial morphology (MitoTracker; scale bar: 50 µm). Mitochondrial reactive oxygen species (MitoSox) in NPCs. Scale bar: 100 µm. K) Quantitative analysis of relative fluorescence intensity of JC‐1 and MitoSox, as well as quantification of mitochondrial fragmentation ratio. *n* = 3, *p < 0.05; **p < 0.01; ***p < 0.001; ****p < 0.0001; ns, not significant. Data are expressed as mean ± standard deviation.

TBHP triggers mitochondrial dysfunction and senescence in NPCs, and treatment with hypoxia‐exo significantly reversed this outcome. To quantify differential gene expression between hypoxia‐ and normoxia‐preconditioned BMSC exosomes, we used the NCBI database (https://www.ncbi.nlm.nih.gov/geo/) for data retrieval. The specific dataset employed in this study was extracted from sample GSE229825, and the processing conditions closely followed those provided by the data submitters. Subsequently, the obtained data were subjected to heat map analysis for visualization (**Figure** **4**A). Gene ontology (GO) and Kyoto Encyclopedia of Genes and Genomes (KEGG) pathway analysis demonstrated that genes within the hypoxia‐induced exosomes were primarily associated with “reactive oxygen species,” “mTOR signaling pathway,” and “autophagy pathway” (Figure 4B), signifying their important role in the hypoxia‐mediated intracellular signaling pathway. Notably, the molecular function enrichment map (Figure 4C) reveals that several hypoxia‐inducible genes were significantly upregulated in the biological process (BP) category. Correspondingly, some mitochondrial membrane, inner and outer mitochondrial membrane, and organelle membrane genes were significantly upregulated in the cell component (CC) category. Furthermore, several oxidoreductase and kinase activity‐related genes were significantly upregulated at the molecular function (MF) level. Thus, hypoxia‐preconditioned extracellular vesicles contain abundant hypoxia‐responsive factors that are closely linked to the mitochondria. To further investigate this attribute, we generated volcano plots (Figure 4D) based on the following: false discovery rate < 0.001 and log FC > 1.5. Among the exocytosis‐related genes upregulated by hypoxia, BNIP3 expression was significantly increased.

To validate the differential expression of BNIP3 among degenerating NPCs, an exogenous degenerating NPC model was induced using TBHP; subsequently, BNIP3 protein expression was verified by western blot analysis (Figure 4E). The results demonstrated significant downregulation of BNIP3 in degenerating NPCs. Additionally, we conducted immunohistochemical and tissue fluorescence analyses of rat tail intervertebral disc slices. Consistent with previous results, we observed higher BNIP3 expression in the hypoxia‐exo‐treated group (Figure S3, Supporting Information). To validate this finding, we simultaneously introduced exogenous hypoxia‐exo and normoxia‐exo into the NPCs. Western blot analysis confirmed that BNIP3 expression increased in NPCs following the addition of hypoxia‐exo compared to BNIP3 levels in the normoxia‐exo‐treated group, thus confirming that hypoxia‐exo upregulated BNIP3 gene expression after endocytosis into NPCs (Figure 4F). Assessment of mitochondrial morphology in TBHP‐treated NPCs using MitoTracker revealed a shortened, flexed phenotype along with a blurred structure and weakened fluorescence intensity, indicating impaired mitochondrial integrity (Figure 4G).

To visualize and confirm the endocytosis of exosomes into NPCs and their interactions, we stained exosome membranes with PKH67 and simultaneously labeled mitochondria in NPCs with MitoTracker (both groups of cells were treated with TBHP). Fluorescence microscopy revealed the co‐localization of exosomes with mitochondria, suggesting their interaction (Figure 4H). Furthermore, we observed that hypoxia‐exos had a higher likelihood of penetrating the NPC membrane than did normoxia‐exos. Moreover, the hypoxia‐exo‐treated group demonstrated superior recovery of mitochondrial morphology and fluorescence intensity compared to that in the normoxia‐exo‐treated group (Figure 4I). Mitochondrial membrane potential was then evaluated using JC‐1, MitoTracker and MitoSox probes in TBHP‐treated NPCs (Figure 4J). Our results revealed a decrease in mitochondrial membrane potential (ΔΨm), with healthy mitochondria showing JC‐1 aggregation in the mitochondrial matrix that formed polymers that emitted strong red fluorescence, while unhealthy mitochondria (PBS‐treated) displayed JC‐1 as a cytosolic monomer producing green fluorescence. MitoTracker detects increased fragmentation of intracellular mitochondria after TBHP treatment, which improves upon addition of Hypoxia‐exo. Additionally, the MitoSox probe showed oxidation by ROS in mitochondria, resulting in intense red fluorescence. However, the addition of hypoxia‐exo resulted in partial restoration of mitochondrial morphology, with a corresponding reversal of the JC‐1 red/green ratio and reduction of mitochondrial ROS. These findings demonstrate the partial rescue of the mitochondrial phenotype after the endocytosis of exosomes by NPCs, leading to reduced mitochondrial fragmentation and oxidative stress damage. Remarkably, the rescue effect was more pronounced in the hypoxia‐exo‐treated group than in the normoxia‐exo‐treated group. Furthermore, we silenced hypoxia‐inducible factor HIF‐1α to illustrate its important role and found that the effect of hypoxia‐exo treatment was partially attenuated, indicating that genes in the exosomes of hypoxia‐pretreated cells are under upstream transcription factor regulation (Figure 4K).

### 2.5 Hypoxia‐Induced Exosome‐Delivered BNIP3 Activates Mitochondrial Autophagy, Alleviating Aging in NPCs

**Figure 5:**
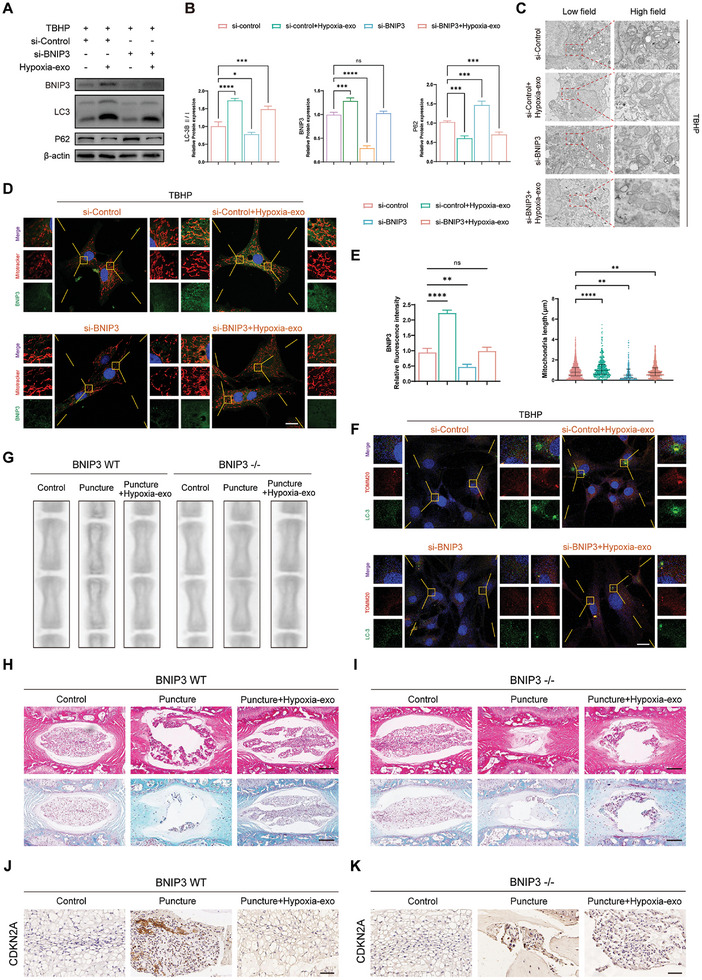
BCL2/adenovirus E1B 19 kDa interacting protein 3 (BNIP3) maintains mitochondrial homeostasis through autophagy activation. A) Western blot analysis of BNIP3, LC3, and P62 expression in wild‐type and BNIP3‐knockout NPCs treated with tert‐butyl peroxide (TBHP) with or without exosomes from hypoxia‐treated cells (hypoxia‐exo). B) Quantification of BNIP3, LC3, and P62 expression. *n* = 3, *p < 0.05; ***p < 0.001; ****p < 0.0001; ns, not significant. C) Mitochondrial morphology changes as observed by transmission electron microscopy. D) Double‐immunofluorescence staining of MitoTracker and BNIP3. Scale bar: 20 µm. E) Quantitative analysis of fluorescence intensity of BNIP3 and mitochondrial length measurements in different treatment groups. *n* = 3, **p < 0.01; ****p < 0.0001; ns, not significant. F) Immunofluorescence detection of changes in the fluorescence intensity of LC3 and translocase of outer mitochondrial membrane 20 (TOMM20) in nucleus pulposus cells (NPCs) after different treatments. Scale bar: 20 µm. G) X‐ray images of BNIP3 wild type (BNIP3 WT) and BNIP3‐/‐ mice after different treatments. H,I) Hematoxylin and eosin (HE) and Safranin‐O (SO) staining of BNIP3 WT and BNIP3‐/‐ mice. Scale bar: 1 mm. J,K) Immunohistochemical analysis of cyclin dependent kinase inhibitor 2A (CDKN2A) expression in BNIP3 WT and BNIP3‐/‐ mice. Scale bar: 200 µm. Data are expressed as mean ± standard deviation.

We quantified autophagic flux in NPCs treated with TBHP using western blot analysis. Moreover, we generated NPCs with reduced BNIP3 levels using a targeted BNIP3 siRNA (si‐BNIP3) approach. Subsequently, we supplemented wild‐type NPCs with hypoxia‐exo and observed a remarkable increase in BNIP3 protein expression concomitant with an increase in the LC3II/I ratio, a crucial marker of autophagy (**Figure** **5**A). Subsequently, BNIP3 knockdown led to a notable decline in autophagic flux and a concurrent increase in substrate release resulting from accumulated P62. Following this, we introduced hypoxia‐exo into BNIP3 knockdown NPCs, resulting in the restoration of BNIP3 expression and subsequent recovery of autophagic flux (Figure 5B). We conducted a comprehensive assessment of the impact of BNIP3 using TEM. The results revealed that in the si‐Control+TBHP group, mitochondria displayed swelling, loss of cristae, and vacuole‐like degeneration (Figure 5C). This phenomenon was ameliorated upon addition of hypoxia‐exo. Subsequently, upon BNIP3 knockdown and exposure to TBHP, mitochondrial damage was notably more pronounced compared to that in the si‐control group. Additionally, supplementation of hypoxia‐exo to the BNIP3 knockout group resulted in restoration of impaired mitochondrial morphology. To further evaluate the effect of this process on NPCs, we performed double‐immunofluorescence staining using MitoTracker and BNIP3 (Figure 5D). In wild‐type NPCs, hypoxia‐exo treatment significantly ameliorated the aberrant mitochondrial morphology induced by TBHP, restoring the rod‐like elongated mitochondrial structure. Furthermore, the fluorescence intensity of MitoTracker staining was enhanced, and a significant increase in BNIP3 fluorescence intensity was observed compared to baseline levels. To ascertain the involvement of BNIP3 in mitochondrial autophagy, we induced mitochondrial damage by adding TBHP subsequent to BNIP3 knockdown. The results demonstrated further deterioration of mitochondrial morphology in BNIP3‐knockdown NPCs compared to that in wild‐type NPCs. However, upon administration of hypoxia‐exo, successful delivery of BNIP3 within the exosomes to the NPCs was observed, leading to the upregulation of BNIP3 fluorescence intensity and partial restoration of mitochondrial morphology (Figure 5D,E). To further investigate this phenomenon, immunofluorescence was used to label the translocase of outer mitochondrial membrane 20 (TOMM20) along with LC3 in NPCs. Upon TBHP‐induced mitochondrial damage, TOMM20 was recruited to the outer mitochondrial membrane and subsequently activated. Research indicates that activation of TOMM20 recruits parkin, leading to the ubiquitination of outer membrane proteins. The ubiquitinated products bind to LC3 and are enclosed in autophagic vesicles.^[^ ^19^ ^]^ By incorporating hypoxia‐exo, the fluorescence intensity and co‐localization of TOMM20 with LC3 were further increased, indicating the successful activation of mitochondrial autophagy (Figure 5F).

To investigate the role of BNIP3 in IVDD, we compared the imaging and histological scores of BNIP3 wild‐type (BNIP3 WT) and BNIP3 knockout (BNIP3‐/‐) mice in both the control and puncture groups at four weeks post‐surgery. The results showed that the NP tissue integrity and intervertebral space height were both lower in the BNIP3‐/‐ group compared to that in the BNIP3 WT group (Figure 5G). Quantitative analysis results are provided in the supplementary materials (Figure S5, Supporting Information). Additionally, following IVDD surgery, the postoperative repair capacity of the BNIP3‐/‐ group was significantly reduced. However, the integrity of intervertebral disc tissue was improved after exogenous addition of hypoxia‐exo (Figure 5H,I). Afterward, we conducted immunohistochemistry to assess the levels of age‐related proteins (CDKN1A, CDKN2A, and TP53). The results revealed mild signs of aging in the NP of BNIP3‐/‐ mice, which became more pronounced following IVDD surgery. Addition of exogenous hypoxia‐exo subsequently partially mitigated the elevation of aging marker proteins (Figure 5J,K). The immunohistochemistry results for TP53 and CDKN1A were consistent with the previous findings (Figure S5, Supporting Information).

To further elucidate the relationship between BNIP3 and mitochondrial autophagy activation in the regulation of human NPC aging, we induced the aging phenotype of NPCs using TBHP and then applied 3‐methyladenine (3‐MA) to inhibit autophagosome formation. We found that 3‐MA treatment exerts beneficial effects on TBHP‐induced aging of NPCs (wild‐type), as evidenced by western blotting analysis of TP53, CDKN1A, and CDKN2A expression and cell staining with β‐gal and EdU (Figures S6 and S7, Supporting Information). Concurrently, MitoTracker analysis revealed an increase in TBHP‐induced mitochondrial fragmentation, which was alleviated by 3‐MA treatment (Figure S7, Supporting Information). In contrast, 3‐MA treatment had deleterious effects on NPCs overexpressing BNIP3 (oe‐BNIP3), exacerbating the aging phenotype compared with that in wild‐type cells. Furthermore, cell proliferation decreased and mitochondrial fragmentation increased in oe‐BNIP3 NPCs treated with 3‐MA (Figure S7, Supporting Information). This suggests that mitochondrial autophagy can be induced by TBHP in both wild‐type and oe‐BNIP3 NPCs and that BNIP3 plays a beneficial role in promoting mitochondrial autophagy, which is crucial for alleviating mitochondrial dysfunction and cellular aging.

### 2.6 The Mitophagy Receptor BNIP3 Interacts with ANXA2 in NPCs, Facilitating the Nuclear Translocation of TFEB

**Figure 6:**
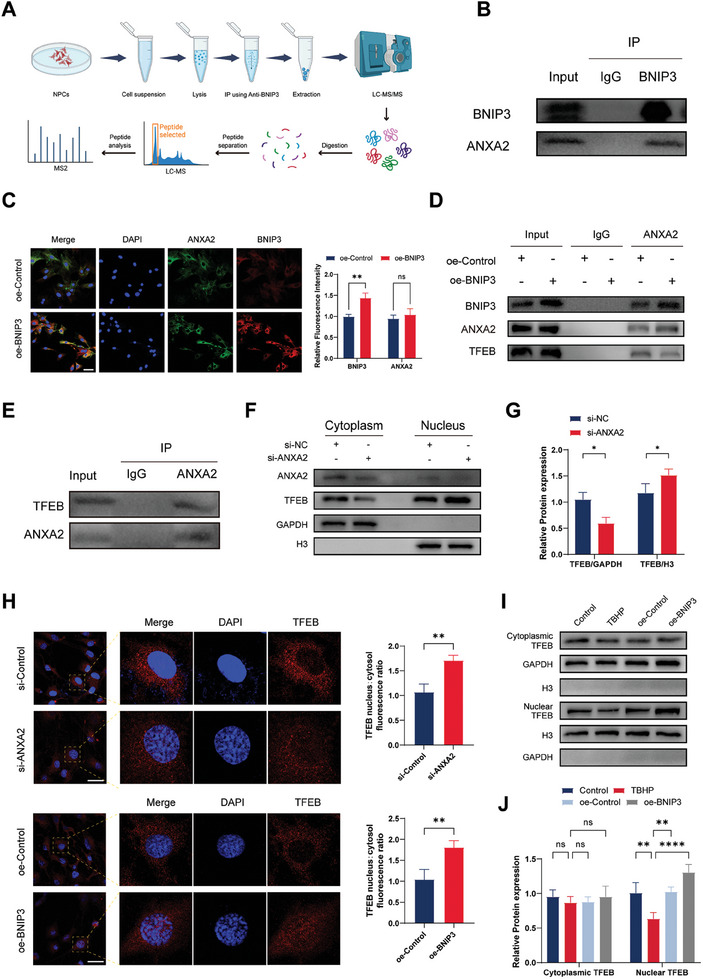
The BCL2/adenovirus E1B 19 kDa interacting protein 3 (BNIP3)–annexin A2 (ANXA2) interaction influences transcription factor EB (TFEB) localization. A) Schematic representation of co‐immunoprecipitation (CO‐IP) and mass spectrometry. B) BNIP3 interaction with ANXA2 demonstrated by CO‐IP. *n* = 3. C) Differential expression of BNIP3 and ANXA2 following overexpression or empty vector expression assessed by immunofluorescence staining analysis. *n* = 3, scale bar: 50 µm. D) Nucleus pulposus cells (NPCs) were transfected with either an BNIP3 plasmid to overexpress BNIP3 or an empty plasmid, followed by CO‐IP to validate the reciprocal binding between BNIP3, ANXA2, and TFEB. *n* = 3. E) CO‐IP experiment confirming TFEB binding to ANXA2. *n* = 3. F) NPCs were subjected to ANXA2 knockdown, and subsequent western blot analysis was employed to examine the cytoplasmic and nuclear expression levels of ANXA2 and TFEB proteins. *n* = 3. G) Quantitative analysis of cytoplasmic and cytosolic TFEB expression was performed. *n* = 3, *p < 0.05. H) In NPCs, ANXA2 was either silenced or BNIP3 was overexpressed, and the spatial localization of TFEB was assessed using immunofluorescence. *n* = 3, scale bar: 50 µm, **p < 0.01. I) Spatial changes in TFEB expression were analyzed by western blot analysis following treatment with tert‐butyl peroxide (TBHP) or an overexpression control plasmid (oe‐control) or overexpression of BNIP3 (oe‐BNIP3) in NPCs. J) Quantification of cytoplasmic and nuclear TFEB expression. *n* = 3, **p < 0.01; ****p < 0.0001; ns, not significant. Data are expressed as mean ± standard deviation.

We investigated the potential mechanisms underlying the involvement of BNIP3 in mitophagy. To validate the potential downstream targets of BNIP3, co‐immunoprecipitation (CO‐IP) and mass spectrometry (MS) were employed to identify the downstream effector proteins associated with BNIP3‐mediated autophagy activation (**Figure** **6**A). We performed a range of analyses, including GO, KOGs, KEGG, IPR, and subcellular localization analyses, on the coprecipitated material to obtain additional information on these downstream effectors (Figure S8, Supporting Information). In summary, annexin A2 (ANXA2) was chosen as a protein that strongly interacts with BNIP3 from a pool of ten potential candidates based on protein abundance analysis and was therefore used for further validation (Table S3, Supporting Information). We validated the protein–protein interactions between BNIP3 and ANXA2 using CO‐IP analysis (Figure 6B). ANXA2 is a calcium‐dependent membrane‐binding protein that plays a crucial role in membrane trafficking and fusion.^[^ ^20^ ^]^ In nonalcoholic fatty liver disease, ANXA2 obstructs autophagic flux mediated by the AMP‐activated protein kinase (AMPK)/mechanistic target of rapamycin (mTOR) pathway.^[^ ^21^ ^]^ However, we observed no significant change in the fluorescence intensity of ANXA2 when BNIP3 expression was upregulated in NPCs (Figure 6C). Hence, we hypothesized that BNIP3 regulates autophagy via an indirect interaction with ANXA2.

Previous studies have indicated that ANXA2 serves as a direct target of bleomycin in interstitial pulmonary fibrosis, forming a complex with transcription factor EB (TFEB) and tyrosine 3‐monooxygenase/tryptophan 5‐monooxygenase activation protein (YWHA) in the cytoplasm, resulting in the cytoplasmic retention of TFEB.^[^ ^20^ ^]^ TFEB has been reported to be an autophagy gene involved in lysosome biogenesis and autophagolysosome regulation. Inhibition of the nuclear translocation of TFEB suppresses lysosomal gene activation and initiation of mitochondrial autophagic flux. Therefore, we conducted immunoprecipitation experiments to determine whether ANXA2 is involved in inhibiting the nuclear translocation of TFEB. The results revealed an interaction between ANXA2 and TFEB (Figure 6E). To verify this interaction, we overexpressed BNIP3 in NPCs and preserved the interacting proteins. Subsequently, we validated this interaction using western blot analysis. The results demonstrated that the interaction between BNIP3 and ANXA2 was enhanced by BNIP3 overexpression, whereas the interaction between ANXA2 and TFEB was weakened (Figure 6D). Next, siRNA (si‐ANXA2) was used to suppress intracellular ANXA2 expression. Western blot analysis of nuclear and cytoplasmic proteins revealed that silencing ANXA2 downregulated the cytoplasmic expression of TFEB and upregulated its nuclear expression. (Figure 6F,G). Similarly, we treated NPCs with si‐ANXA2 and performed immunofluorescence analysis. The results revealed that, compared to the negative control cells (si‐control), cells treated with si‐ANXA2 displayed enhanced nuclear expression of TFEB (Figure 6H). These results indicate that ANXA2 forms a complex with TFEB in the cytoplasm, preventing TFEB from entering the nucleus and becoming deactivated. Subsequently, we overexpressed BNIP3 in NPCs, and immunofluorescence analysis revealed that increased levels of BNIP3 promoted the nuclear translocation of TFEB. Similarly, we performed western blot analysis after separating nuclear and cytoplasmic proteins and observed that oe‐BNIP3 in NPCs did not result in a significant difference in cytoplasmic TFEB expression compared to that in the control group. However, an upward trend in nuclear TFEB protein levels compared to those in the control group was observed (Figure 6I,J). In conclusion, we established an association between BNIP3 and ANXA2 in NPCs, which reduced the interaction between ANXA2 and TFEB and promoted the nuclear translocation of TFEB.

### 2.7 TFEB is a Pivotal Regulator of Lysosomal Biogenesis and Function

**Figure 7:**
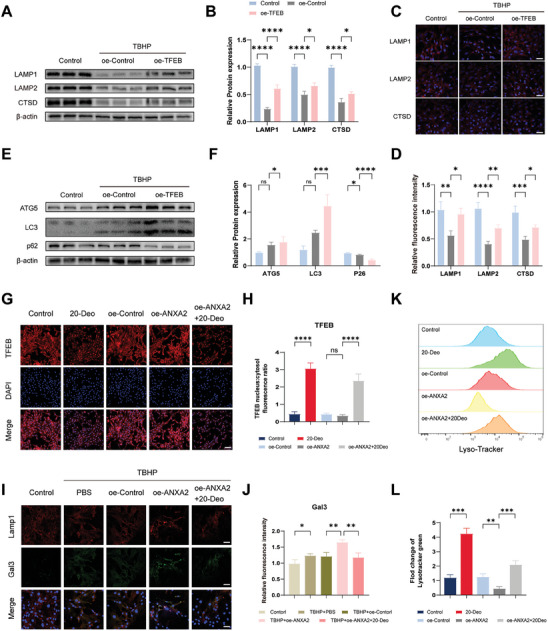
The influence of TFEB abundance and subcellular localization on lysosomal function. A) Expression analysis of lysosomal associated membrane protein (LAMP)−1, LAMP2, and cathepsin D (CTSD) using western blot analysis. B) Quantitative assessment of relative protein levels of LAMP1, LAMP2, and CTSD. *n* = 3, *p < 0.05, ****p < 0.0001. C) Fluorescence intensity detection of LAMP1, LAMP2, and CTSD by immunofluorescence assay. Scale bar: 50 µm. D) Quantitative analysis of LAMP1, LAMP2, and CTSD using immunofluorescence. *n* = 3, *p < 0.05, **p < 0.01, ***p < 0.001. E) Expression analysis of LC3, autophagy related 5 (ATG5), and P62 using western blot analysis. F) Protein quantification analysis of LC3, ATG5, and P62. *n* = 3, *p < 0.05; **p < 0.01; ****p < 0.0001; ns, not significant. G) Spatial distribution analysis of TFEB after different treatments using immunofluorescence. Scale bar: 100 µm. H) Quantitative analysis of nuclear/cytoplasmic protein ratio of TFEB. *n* = 3, ****p < 0.0001; ns, not significant. I) Immunofluorescence staining for LMAP1 and galectin‐3 GAL3 after different treatments. Scale bar: 50 µm. J) Quantitative analysis of GAL3 fluorescence intensity. *n* = 3, *p < 0.05, **p < 0.01. K) Treatment with 20‐deoxyingenol enhances TFEB nuclear translocation to augment Lyso Tracker Red staining. L) Quantitative analysis of the relative changes in Lyso Tracker Red. **p < 0.01; ***p < 0.001. Data are expressed as mean ± standard deviation.

During mitophagy, the degradation and recycling of mitochondria depend on gene regulation within the lysosome. We further speculated that TFEB expression may enhance autophagic flux in NPCs, thereby activating the expression of lysosome‐associated genes. Through western blot analysis of the lysosomal markers lysosomal associated membrane protein (LAMP)−1 and LAMP2 and lysosomal protease cathepsin D (CTSD), we observed a reduction in lysosomal protein expression in NPCs under TBHP treatment (**Figure** **7**A). In NPCs overexpressing TFEB, TFEB upregulation promoted the expression of the lysosome‐associated proteins LAMP1, LAMP2, and CTSD (Figure 7B). This finding was further confirmed by immunofluorescence analysis (Figure 7C,D). Subsequently, we evaluated the autophagic function mediated by TFEB and found that TFEB overexpression increased autophagy related 5 (ATG5) and LC3B‐II expression, while reducing the levels of the substrate P62 (Figure 7E,F).

To further explore the influence of TFEB and its subcellular localization on gene function, we conducted experiments using 20‐deoxyingenol (20‐Deo), which induces the translocation of TFEB to the nucleus, thereby enhancing autophagy and lysosomal biogenesis. NPCs were subjected to different treatments, including PBS, 20‐Deo, overexpression of a control plasmid (oe‐control), overexpression of ANXA2 (oe‐ANXA2), and oe‐ANXA2+20‐Deo, to assess the changes in autophagic flux within the NPCs. Immunofluorescence results revealed that compared to levels in the control group, treatment with 20‐Deo significantly facilitated the nuclear expression of TFEB, whereas no significant difference was observed between the oe‐control‐treated and control groups. Furthermore, oe‐ANXA2 reduced the nuclear translocation of TFEB, which was partially restored by exogenous addition of 20‐Deo (Figure 7G,H).

Galectin‐3 (GAL3) is a specific marker of lysosomal and endolysosomal membrane damage and has been extensively employed in the investigation of lysosomal membrane injury.^[^ ^22^ ^]^ We conducted a co‐localization immunofluorescence analysis of GAL3 and LAMP1 (a lysosomal membrane protein) to examine these phenomena. We found that the addition of TBHP induced lysosomal damage, leading to an increase in GAL3 levels (Figure 7I,J). Additionally, when ANXA2 was overexpressed in NPCs and TBHP was introduced, a further increase in the expression of GAL3 was observed. The subsequent administration of 20‐Deo to ANXA2‐overexpressing NPCs partially reduced the fluorescence intensity of GAL3.

Next, we utilized flow cytometry to assess the lysosomal acidification fluorescent probe, Lyso Tracker. The results revealed that 20‐Deo facilitates TFEB nuclear translocation, thereby enhancing the biological occurrence of Lysotracker (Figure 7K,L). Conversely, overexpression of ANXA2 led to a reduction in Lysotracker intensity by inhibiting TFEB nuclear translocation. These results indicate that nuclear translocation of TFEB effectively increases the number of acidified lysosomes, promoting lysosomal biogenesis.

### 2.8 The BNIP3/ANXA2 Axis Regulates IVDD Progression in Rats

**Figure 8:**
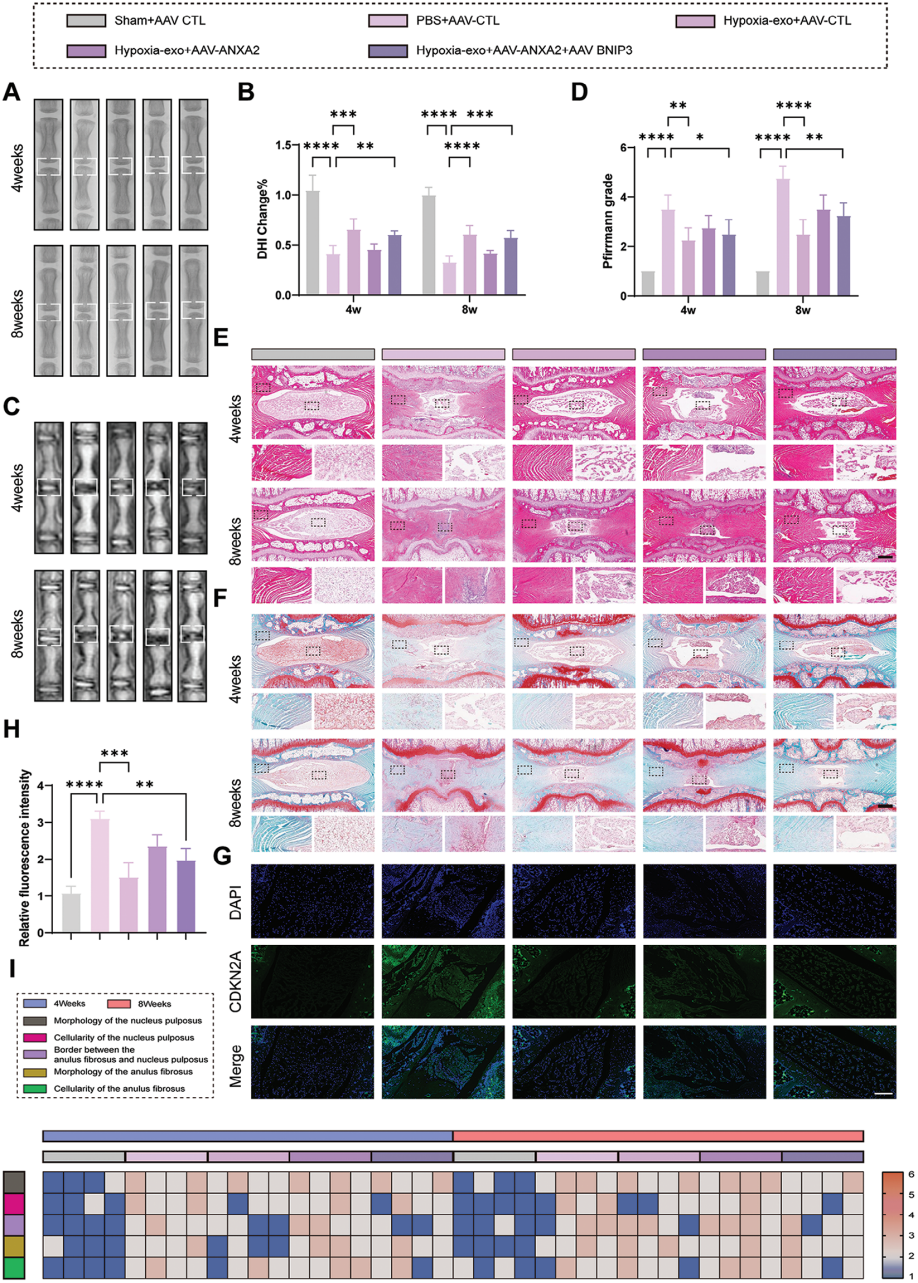
The BCL2/adenovirus E1B 19 kDa interacting protein 3 (BNIP3)/annexin A2 (ANXA2) axis regulates the progression of intervertebral disc degeneration (IVDD) in vivo. A) X‐ray images of rat tail vertebrae were taken at 4 and 8 w post‐injection with adenoviral‐associated viruses (AAVs). B) Quantitative analysis of rat disc height index scores. *n* = 4, **p < 0.01; ***p < 0.001; ****p < 0.0001; C) Magnetic resonance imaging (MRI) with T2‐weighted sequences was conducted at 4 and 8 w post‐injection with AAVs. D) Quantitative analysis of Pfirrmann grades. *n* = 4, *p < 0.05; **p < 0.01; ***p < 0.001; ****p < 0.0001; E) Hematoxylin and eosin (HE) staining of rat tail vertebrae slices after different treatments. Scale bar: 1 mm. F) Safranin‐O/Fast Green (SO) staining of rat tail vertebrae slices after different treatments. Scale bar: 1 mm. G) Fluorescent staining images of cyclin dependent kinase inhibitor 2A (CDKN2A) in rat tail vertebrae slices from each experimental group. Scale bar: 200 µm. H) Quantitative analysis of the relative fluorescence intensity of CDKN2A in each experimental group. *n* = 4, ***p < 0.001; ****p < 0.0001; I) Plotting histological scores of each experimental group in heatmap format. *n* = 4. Data are expressed as mean ± standard deviation.

To validate the efficacy of ANXA2 and BNIP3 in vivo in rats, we established a rat IVDD model and introduced ANXA2 and BNIP3 adenoviral‐associated viruses (AAVs) into NPCs through disc injection. Four weeks post‐surgery, X‐ray imaging and DHI analysis revealed a significant degenerative trend in the PBS+AAV‐control (CTL) group compared to the Sham+AAV‐CTL group. However, the addition of exogenous hypoxia‐exo showed certain improvements in the intervertebral space. Notably, co‐administration of hypoxia‐exo and AAV‐ANXA2 partially counteracted the efficacy of the exosomes (**Figure** **8**A,B). MRI T2‐weighted images of the rat intervertebral discs revealed a reduction in intervertebral space and decreased NPC hydration in the PBS+AAV‐CTL group compared to the Sham group. However, treatment with hypoxia‐exo+AAV‐CTL showed the most favorable therapeutic effects, superior to treatment with hypoxia‐exo+AAV‐ANXA2. Nevertheless, exogenous supplementation of AAV‐BNIP3 rescued the negative effects induced by AAV‐ANXA2 (Figure 8C,D). Furthermore, HE and SO staining revealed a substantial disappearance of NPCs in the PBS+AAV CTL group, with a more pronounced degeneration observed at eight weeks compared to four weeks post‐treatment (Figure 8E,F). Conversely, the hypoxia‐exo+AAV‐CTL group exhibited the most favorable therapeutic efficacy, while the addition of AAV‐ANXA2 attenuated the NP repair capability. Addition of AAV‐BNIP3 promoted ECM synthesis in the NPCs while inhibiting its degradation. We then conduced histological evaluations of the morphology of the NP, cellularity of the NP, border between the AF and NP, morphology of the AF, and cellularity of the AF and presented the results in heat maps (Figure 8I). Tissue fluorescence analysis of CDKN2A content revealed significant aging phenotypes in the PBS+AAV CTL group compared to the Sham+AAV‐CTL group. Treatment with hypoxia‐exo demonstrated the most effective rescue of aging in NPCs. In the hypoxia‐exo+AAV‐ANXA2 group, partial defects were observed in the NP tissue, accompanied by increased expression of CDKN2A (Figure 8G,H). Fluorescence results of CDKN1A and TP53 after different treatments are provided in the supplementary materials (Figure S9, Supporting Information).

## 3 Discussion

Treatment of IVDD, an irreversible form of cellular aging, is primarily focused on alleviating the associated symptoms of lower back and leg pain but remains a challenge. Current therapeutic approaches mainly involve nonpharmacological interventions, pharmacotherapy, and surgical procedures.^[^ ^23^ ^]^ However, relying solely on nonsteroidal anti‐inflammatory drugs or total disc excision surgery is insufficient to effectively reverse the degenerative trend and may even lead to more severe consequences.^[^ ^24^ ^]^ Therefore, cellular and molecular approaches that target the pathogenesis and underlying mechanisms of this condition are highly desirable. Moreover, the inevitable senescence and limited proliferative capacity of NPCs are significant factors contributing to the compromised integrity of NP tissues.^[^ ^25^ ^]^ Cellular senescence, along with the inflammatory response mediated by the senescence‐associated secretory phenotype, is a critical factor contributing to the progressive deterioration of the intervertebral disc. In this study, we initially observed a significant upregulation of aging‐related protein markers in degenerated NP tissues accompanied by impaired mitochondrial function. To investigate this further, we established an in vitro model of NP degeneration using TBHP.^[^ ^26^ ^]^

Hypoxia restricts the cellular susceptibility to oxidative DNA damage and genetic mutations.^[^ ^27^ ^]^ Hence, hypoxia is considered a crucial factor for enhancing cellular function, and we therefore aimed to optimize the state of stem cells through hypoxic preconditioning. Research suggests that hypoxia‐preconditioned exosomes derived from BMSCs exhibit superior therapeutic efficacy in osteoarthritis compared to the those in an untreated group.^[^ ^6^ ^]^ The gain‐of‐function of paracrine secretion induced by stem cell preconditioning has positioned exosome preconditioning as a promising therapeutic approach. Following a simple, effective, and easily implementable method, we cultured BMSCs in a 2% hypoxic incubator, extracted the extracellular vesicles, and revealed a novel mechanism of mitochondrial autophagy mediated by the HIF‐1α‐BNIP3‐ANXA2 axis through differential gene enrichment analysis. As a key regulator of the hypoxic response, HIF‐1α undergoes proteasomal degradation via prolyl hydroxylation under physiological conditions.^[^ ^28^ ^]^ Additionally, HIF‐1α is subjected to multiple post‐translational modifications, including acetylation and phosphorylation.^[^ ^29^ ^]^ Under conditions of oxygen deprivation, the activity of hydroxylases is suppressed, leading to the accumulation of HIF‐1α. Subsequently, HIF‐1α binds to hypoxia response elements within the cells, thereby controlling the transcription and regulation of downstream genes.^[^ ^30^ ^]^ Similarly, the decrease in mitochondrial membrane potential (ΔΨm), which is negatively regulated under hypoxia, inhibits the accumulation of cytochrome c and the activation of caspase‐3 and 9, thereby effectively restoring a stable mitochondrial membrane potential state.^[^ ^31^ ^]^

Four outer mitochondrial membrane proteins, FUN14 domain containing 1 (FUNDC1), BCL2 like 13 (BCL2L13), NIX, and BNIP3, are direct receptors for mitophagy.^[^ ^32^ ^]^ As a member of the BCL‐2 family, BNIP3 is widely localized in the outer mitochondrial membrane and exhibits high sensitivity to HIF‐1α.^[^ ^29^ ^]^ In this study, we demonstrated for the first time that hypoxia triggers the upregulation of HIF‐1α in mesenchymal stem cells, resulting in increased BNIP3 expression and its packaging into secreted exosomes. Exogenously delivered BNIP3 rescued the downregulation of BNIP3 caused by IVDD. In aging NPCs, the lack of BNIP3 limited the activation of mitochondrial autophagic flux, which crucially regulates the initiation of mitochondrial autophagy through independent or tandem receptor activation pathways.

ANXA2 is a calcium‐dependent membrane‐binding protein that exists in cells as a monomer or heterotetramer. Monomers are present in the cytoplasm, nucleus, and early nucleoli, whereas heterotetramers are present on the cell membrane.^[^ ^33^ ^]^ Recent studies have reported that ANXA2 aggravates hepatic lipid metabolic injury by blocking AMPK/mTOR signaling‐mediated autophagic flux in the liver.^[^ ^21^ ^]^ In addition, ANXA2 can bind to FAM3 metabolism regulation signaling molecule A (FAM3A) and activate Rho GTPase to regulate the AKT serine/threonine kinase 1 (AKT1)‐mTOR‐unc‐51 like autophagy activating kinase (ULK)−1/2 signaling axis.^[^ ^34^ ^]^ TFEB, a member of the MiT‐TEF family of transcription factors, is a critical regulator of lysosomal transcription. It is activated by protein phosphatase 3 (PPP3)/calcineurin‐mediated dephosphorylation during ROS‐mediated lysosomal Ca^2+^ release and regulates the expression of receptor genes optineurin (OPTN) and calcium binding and coiled‐coil domain 2 (CALCOCO2) through nuclear translocation.^[^ ^35^ ^]^ In addition, studies have demonstrated a significant correlation among mitochondrial ROS‐mediated lysosomal Ca^2+^ release, endoplasmic reticulum lysosomal Ca^2+^ filling, and TFEB activation.^[^ ^36^ ^]^ In this study, we conducted the first screen to identify ANXA2 as an interacting partner of BNIP3. The binding of BNIP3 to ANXA2 facilitated the release of TFEB and reduced its cytoplasmic retention. Additionally, this release promoted the direct interaction of TFEB with the ATG family members LC3, p62/SQSTM1.

In summary, we developed a hypoxia treatment strategy for optimizing the bioavailability of stem cell‐derived exosomes in NP tissues. By enriching exosomes with BNIP3, we introduced a novel approach for exogenous autophagy receptor gene delivery. The impact of hypoxia pretreatment on the physicochemical properties of exosomes was negligible. However, further investigation is needed to understand the enhanced characteristics of recipient NPC uptake compared to that of untreated mesenchymal stem cell‐derived exosomes. Furthermore, we validated the biocompatibility of the exosomes by in vivo injection into Sprague‐Dawley rats. These findings demonstrate the significant potential of this novel hypoxia‐induced BMSC‐derived exosome strategy for the future clinical treatment of IVDD.

## 4 Experimental Section

### Human BMSCs

The human mesenchymal stem cell samples used in this study were obtained from patients at the Second Affiliated Hospital of Wenzhou Medical University. This study was approved by the Medical Ethics Committee of the Second Affiliated Hospital of the Wenzhou Medical University (Wenzhou, China; approval number: LCKY2020‐157). The cells were seeded into 25 cm^2^ flat‐bottomed cell culture flasks with BMSC complete medium (basal medium; OriCell, USA) supplemented with 10% fetal bovine serum (FBS; OriCell). The cells were subsequently cultured in an incubator at 37 °C under 5% CO_2_. Passaging was carried out when the cells reached 70–80% confluence.

### Collection of Human Intervertebral Disc Samples

The human NP tissue samples used in this study were procured from patients at the Second Affiliated Hospital of Wenzhou Medical University with the approval of the Medical Ethics Committee of the Second Affiliated Hospital of Wenzhou Medical University (Wenzhou, China; approval number: LCKY2020‐157). All patients provided written, informed consent prior to the procedure. The control group consisted of NP tissue specimens obtained from patients with trauma‐induced lumbar spine fractures (*n* = 4; mean age, 27 years; age range: 24–30 years). The degenerative disc group was further divided into groups II, III, IV, and V based on T2‐weighted images obtained via MRI. The study population comprised patients aged 45–65 years (*n* = 4; mean age, 54 years).

### Animal Model

Sprague‐Dawley rats were selected as experimental animals in this study. All rats were obtained from the Zhejiang Provincial Laboratory Animal Center (Hangzhou, China), and their use was approved by the Laboratory Animal Ethics Committee of Wenzhou Medical University (No. WIUCAS23020208). Rats weighing 250–300 g were selected for needle puncture surgery. The needle gauge 22G was used. Needle punctures were performed at the 7th/8th caudal vertebrae (Co7/8) under radiographic guidance. A needle was inserted into the intervertebral disc space at a fixed depth of 3 mm. After insertion, the needle was rotated 360° and left in place for 30 s. Following the needle puncture procedure, exosomes from the different treatment groups were injected into the central intervertebral disc space using a microinjector. Every 7 days, injections were administered at a dosage of 5 µl, with a concentration of 1 µg µl^−1^. The schematic diagram of the rat acupuncture model and exosome injection can be found in Figure S10 (Supporting Information). Experimental procedures involving animal models and exosome injection can be found in Figure S11 (Supporting Information). Tissue degeneration levels were assessed using the DHI method and graded according to the Pfirrmann grading system, aided by radiography and MRI. The grading was evaluated by three experts specializing in orthopedics.

### Hypoxia Treatment and Exosome Extraction

Cultured BMSCs that reached 70–80% confluence were subjected to a series of procedures. First, the cells were rinsed three times with PBS, and the medium was replaced with basal medium (OriCell) supplemented with 10% exosome‐free serum (OriCell). The cells were then cultured in a low‐oxygen incubator at 37 °C under a gas mixture of 2% O_2_ and 5% CO_2_ for 24 h. After incubation, the cell culture medium was collected. Subsequently, exosomes were extracted using differential ultracentrifugation and purification. The extraction process involved centrifugation at 300×g for 10 min to separate the cells into groups. Dead cells were then removed by centrifugation at 2000×g for 10 min, followed by the removal of cell debris at 10000×g for 30 min. The exosome precipitate was separated by ultracentrifugation to obtain exosome samples. Purification was then performed by washing the exosomes with PBS and subjecting them to ultracentrifugation at 100000×g to remove residual proteins. The exosome surface markers TSG101, HSP70, CD63 and Calnexin were detected using western blot analysis. The morphological characteristics of the exosomes were observed by using TEM (Hitachi, Tokyo, Japan). The sizes of the exosomes were determined by nanoparticle tracking analysis. To verify endocytosis efficiency, exosomes were labeled with PKH26 and PKH67 dyes (Solarbio, Beijing, China) and then internalized into NPCs.

### ROS Measurements

Dihydroethidium (DHE; Beyotime) and DCFH‐DA (Beyotime) staining were used for ROS detection. The final concentration of the working solution was diluted to 10 µmol L^−1^, according to the manufacturer's instructions, and was subsequently added to the NPCs, which were incubated for 20 min at 37 °C. The cells were then removed, washed three times with PBS, and observed and photographed using a confocal microscope (Olympus, Tokyo, Japan).

### Mitochondrial Analysis

NPCs were seeded onto confocal dishes and co‐incubated with MitoTracker Red (Invitrogen) for 30 min. The cells were then observed and imaged using a confocal microscope. JC‐1 (C2006; Beyotime) was used to analyze the membrane potential of living cells. NPCs were labeled with JC‐1 for 30 min, followed by observation and imaging. MitoSox reagent (40778ES50; Yeasen Biotechnology, Shanghai, China) was used to measure superoxide production, and the powder was dissolved into dimethyl sulfoxide to prepare a 5 mm stock solution, which was then diluted to a 5 µm working solution. The resulting working solution was added to a 12‐well plate containing the cells. In addition to MitoSox staining, all cell nuclei were stained with 4′,6‐diamidino‐2‐phenylindole (DAPI; Solarbio, C0065) and visualized using a laser confocal microscope (Olympus).

### β‐Gal Cell Senescence Staining

A Senescence β‐Galactosidase Staining Kit (C0602; Beyotime) was used, according to the manufacturer's instructions, to assess cellular senescence in the fixed NPCs. Specifically, 1 mL of the staining fixative was added to the NPCs, which were then treated and fixed for 15 min at room temperature. Following this, the staining working solution was prepared according to the recommended ratio; NPCS were incubated with this solution overnight at 37 °C. Subsequently, stained cells were observed and counted under a light microscope (Olympus, Tokyo, Japan).

### EdU Cell Proliferation Staining

Cell proliferation was assessed using the Cell‐Light EdU Apollo In Vitro Kit (C10310; Ribobio, Guangzhou, China), which employs EdU labeling. EdU, an analog of thymine deoxyribonucleotide that can substitute thymine deoxyribonucleoside (T) in the cell nucleus during the proliferative phase, was introduced into the treated NPCs. Co‐incubated cells were fixed with 4% paraformaldehyde, washed, and permeabilized. Subsequently, the Apollo staining reaction solution was used for staining. The cells were then incubated at room temperature, shielded from light, and decolorized on a shaker for 30 min. Later, the NPCs were labeled with Hoechst33342 for 30 min, and the resulting images were observed under a fluorescence microscope.

### Western Blotting

Cellular protein extraction was performed by placing cells in a RIPA lysis and extraction buffer supplemented with protease and phosphatase inhibitors (Thermo Fisher Scientific). The protein concentration was normalized using a BCA protein assay kit (Beyotime). Subsequently, 8–12% Sodium dodecyl‐sulfate polyacrylamide gel electrophoresis gels were used for electrophoresis, and the separated proteins were transferred onto polyvinylidene fluoride membranes. The membranes were then sealed using a rapid‐closure solution (Beyotime) at room temperature for 20 min before being incubated with the primary antibody overnight at 4 °C. After incubation with the appropriate secondary antibody at room temperature for 1 h, the membranes were treated with an Enhanced ECL Chemiluminescence Detection Kit (Vazyme, Nanjing, China). For detailed information on the antibodies used, please refer to the Table S4 (Supporting Information).

### Quantitative Real‐Time PCR

NPCs were grown in 6‐well plates, and after different treatments, total RNA was extracted using a Total RNA Extraction Kit (Beyotime). cDNA was synthesized using a PrimeScript RT kit (Takara, Kyoto, Japan) according to the manufacturer's instructions. An RT‐PCR system (LightCycler 96; Roche, Basel, Switzerland) was used for amplification. The RT‐PCR parameters were as follows: 40 cycles of 30 s at 95 °C, 5 s at 95 °C, and 30 s at 60 °C. Primer sequences are available in the Table S5 (Supporting Information).^[^ ^38^ ^]^

### Cell Transfection

To silence HIF‐1α, BNIP3, ANXA2, and TFEB, targeted mRNA and their negative control small interfering RNAs (siRNAs) (Ribbio, China) were transiently transfected, according to the manufacturer's protocol. All the sequences are listed in Table S6 (Supporting Information).

### Immunofluorescence

NPCs were seeded in confocal dishes and subjected to various treatments. Subsequently, the cells were washed three times with PBS and fixed with 4% paraformaldehyde. Cell membranes were permeabilized by treatment with 0.1% Triton X‐100, followed by blocking with 5% goat serum albumin. The primary antibodies used were against MMP13 (#18165‐1‐AP; Proteintech, Rosemont, IL, USA), COL‐II (#ab307674; Abcam, Cambridge, UK), BNIP3 (#sc56167; Santa Cruz Biotechnology, Dallas, TX, USA), TOMM20 (#42 406; Cell Signaling Technology, Danvers, MA, USA), ANXA2 (#11256‐1‐AP; Proteintech), and TFEB (#13372‐1‐AP; Proteintech). The cells were incubated with primary antibodies and subsequently treated with FITC‐labeled goat anti‐rabbit or anti‐mouse IgG (Beyotime) or Cy3‐labeled goat anti‐rabbit or anti‐mouse IgG (Beyotime). DAPI (Solarbio, C0065) was used to stain cell nuclei.

### Co‐IP Followed by MS

A co‐immunoprecipitation (Co‐IP) kit (Thermo Fisher Scientific) was used to isolate protein complexes. The lysate was subjected to gentle centrifugation and incubated overnight at 4 °C with the primary antibody targeting the protein of interest, BNIP3, following the manufacturer's instructions. The next day, antigen/antibody complexes were captured using Pierce protein A/G beads (Thermo Fisher Scientific) and incubated for 1 h at room temperature. The beads were then washed twice with IP lysis/washing buffer and once with purified water. The antigen/antibody complexes were eluted and subjected to western blot analysis. The eluted products were further analyzed using an EASY‐nLC liquid chromatography system with an HFX mass spectrometer (Thermo Fisher Scientific), and the resulting raw files were directly imported into the Proteome Discoverer 2.5 software (Thermo Fisher Scientific) for database searching, peptide identification, and protein quantification.

### X‐Ray and MRI Examinations

X‐ray images of rat tails were obtained before surgery and 4 and 8 weeks after surgery using a XPERT.8 X‐ray machine (Kubtec, Stratford, CT, USA). DHI was determined from the captured images according to the methodology outlined in the Appendix. Additionally, weighted MRI of the rat tails was conducted 4 and 8 w after surgery using an Achieva 3.0 T MRI system (Philips, Amsterdam, The Netherlands). The evaluation and analysis of the images focused on the high‐signal‐intensity region corresponding to the NP on T2‐weighted images in the sagittal plane. The degree of IVDD was assessed by a panel of more than three orthopedic fellows. They measured and evaluated the IVDD based on predetermined criteria.

### Histological Analysis

Human‐derived NP tissues were obtained and fixed in 4% paraformaldehyde (PFA). Following fixation, the tissue was dehydrated, embedded in paraffin, and sectioned into blocks. Immunofluorescence staining was performed on sections for further analysis. Rat intervertebral disc tissue was harvested from Co7/8 of the rat tails. The collected caudal vertebral tissues were fixed with PFA, followed by decalcification using 10% Ethylenediamine tetraacetic acid for 8 w. After decalcification, the tissues were dehydrated, paraffin‐embedded, and sectioned to a thickness of 5 µm. To observe morphological changes in the medulla, surrounding fibrous ring, and cartilage endplates, the sections were subjected to hematoxylin and eosin (HE), Safranin‐O/Fast Green (SF), and immunohistochemical (IHC) staining procedures.

### Statistical Analysis

All data were presented as mean ± standard deviation. Statistical analyses were performed using the Prism 9 software (GraphPad Software, La Jolla, CA, USA). To assess group differences, the data were subjected to statistical analysis using a two‐tailed Student's t‐test or one‐way analysis of variance, followed by a post‐hoc Tukey's multiple comparison test. Statistical significance was set at *p < 0.05, **p < 0.01, ***p < 0.001, and ****p < 0.0001. “ns” denotes non‐significant differences.

### Ethics Approval Statement

The human experiments in this study were approved by the Medical Ethics Committee of the Second Affiliated Hospital of the Wenzhou Medical University (Wenzhou, China; approval number: LCKY2020‐157). The animal experiments in this study were approved by the Laboratory Animal Ethics Committee of Wenzhou Medical University (No. WIUCAS23020208).

## Conflict of Interest

The authors declare no conflict of interest.

## Author Contributions

Y.J., O.W., and Q.C. contributed equally to this work. Y.J., O.W, and Q. C. performed investigation, data curation, methodology, and wrote the original draft. L.C. and Z.Z. performed methodology and software. H.T., H.Z., and K.Z. performed conceptualization and investigation. J.G., X.W., Z.G., and J.S. performed formal analysis and validation. K.K., M.J., Y.L., and X. W. performed supervision and visualization. E.Z. and P.M. performed wrote the original draft, and wrote, reviewed, and edited the draft. S.S. performed methodology and supervision. A.W. performed project administration, supervision, and provided resources.

## Supporting information

Supporting Information

## Data Availability

The data that support the findings of this study are available from the corresponding author upon reasonable request.
