# Supplementary material for: Hypoxia‐Preconditioned BMSC‐Derived Exosomes Induce Mitophagy via the BNIP3–ANAX2 Axis to Alleviate Intervertebral Disc Degeneration
Source: Adv Sci (Weinh). 2024 Jul 8;11(34):2404275. doi: 10.1002/advs.202404275 (PMC11425632; doi:10.1002/advs.202404275)
Supplement: Supplementary file 1 — Supporting Information [file ADVS-11-2404275-s001.docx]

Supporting Information

**Hypoxia-preconditioned BMSC-derived Exosomes Induce Mitophagy via the BNIP3–ANAX2 Axis to Alleviate Intervertebral Disc Degeneration**

Yuxin Jin^1, #^, Ouqiang Wu^1, #^, Qizhu Chen^1, #^, Linjie Chen^1^, Zhiguang Zhang^1^, Haijun Tian^2^, Hao Zhou^1^, Kai Zhang^3^, Jianyuan Gao^1^, Xinzhou Wang^1^, Zhenyu Guo^1^, Jing Sun^1^, Kenny Yat Hong Kwan^4^, Morgan Jones^5^, Yan Michael Li^6^, Ehsan Nazarzadeh Zare^7^, Pooyan Makvandi^8,9^, Xiangyang Wang^1^, Shuying Shen^10*^, Aimin Wu^1*^

^1^Department of Orthopaedics, Key Laboratory of Structural Malformations in Children of Zhejiang Province, Key Laboratory of Orthopaedics of Zhejiang Province, The Second Affiliated Hospital and Yuying Children's Hospital of Wenzhou Medical University, Wenzhou, Zhejiang, 325000, China

^2^Department of Orthopaedic Surgery, Shanghai Sixth People's Hospital Affiliated to Shanghai Jiao Tong University School of Medicine

^3^Shanghai Key Laboratory of Orthopedic Implants, Department of Orthopedics, Ninth People's Hospital, Shanghai Jiao Tong University School of Medicine, Shanghai, China

^4^Department of Orthopaedics and Traumatology, Li Ka Shing Faculty of Medicine, The University of Hong Kong, 5/F Professorial Block, Queen Mary Hospital, 102 Pokfulam Road, Pokfulam, Hong Kong

^5^Spine Unit, The Royal Orthopaedic Hospital, Bristol Road South, Northfield, Birmingham, B31 2AP, UK

^6^Department of Neurosurgery, University of Rochester Medical Center, Rochester, New York, 601 Elm-wood Ave, Rochester, NY 14642

^7^School of Chemistry, Damghan University, Damghan, 36716-45667, Iran

^8^Institute for Bioengineering, School of Engineering, The University of Edinburgh, Edinburgh, UK

^9^Chitkara Centre for Research and Development, Chitkara University, Himachal Pradesh-174103 India

^10^Department of Orthopaedics, Key Laboratory of Musculoskeletal System Degeneration and Regeneration Translational Research of Zhejiang Province, Sir Run Shaw Hospital, Zhejiang University School of Medicine, Hangzhou, 310000, China

^#^These authors contributed equally: Yuxin Jin, Ouqiang Wu, Qizhu Chen

*Corresponding authors: Aimin Wu, Shuying Shen,

*e-mail: aiminwu@wmu.edu.cn, 11207057@zju.edu.cn

**SUPPLEMENTARY FIGURES**


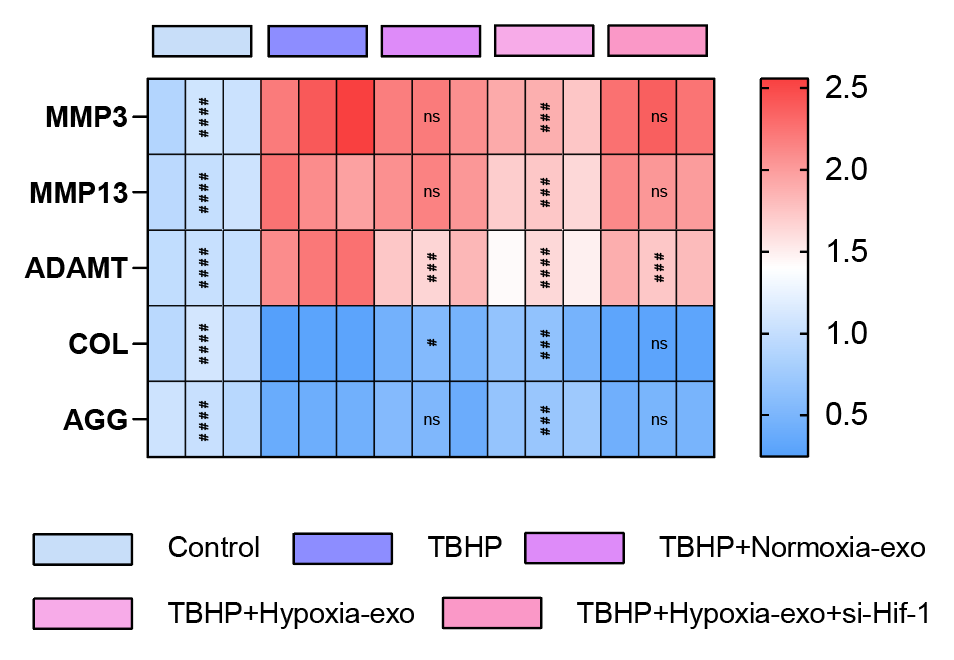


**Figure S1.** qPCR results of exosomes alleviating nucleus pulposus extracellular matrix; #*p* <0.05, ## *p* <0.01, ### *p* <0.001 VS #### *p* <0.0001, ns not statistically vs TBHP.


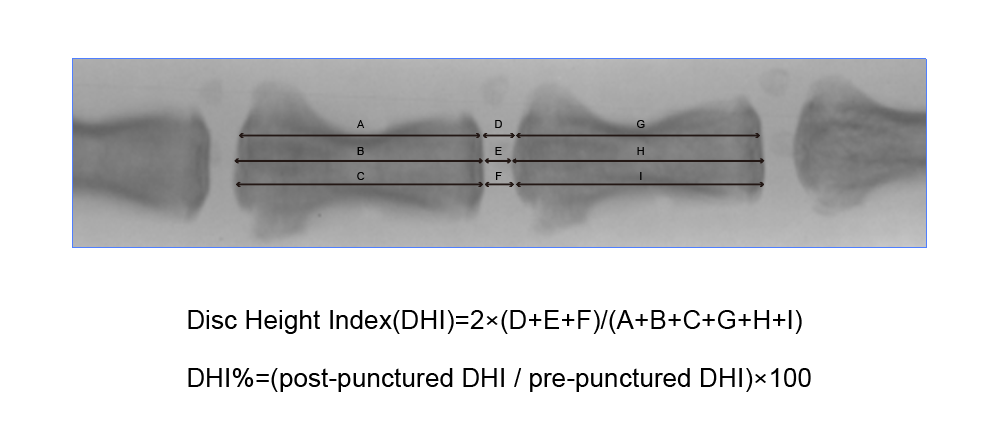


**Figure S2.** Measurement of Disc Height Index (DHI) for Intervertebral Space Height in X-ray Images.


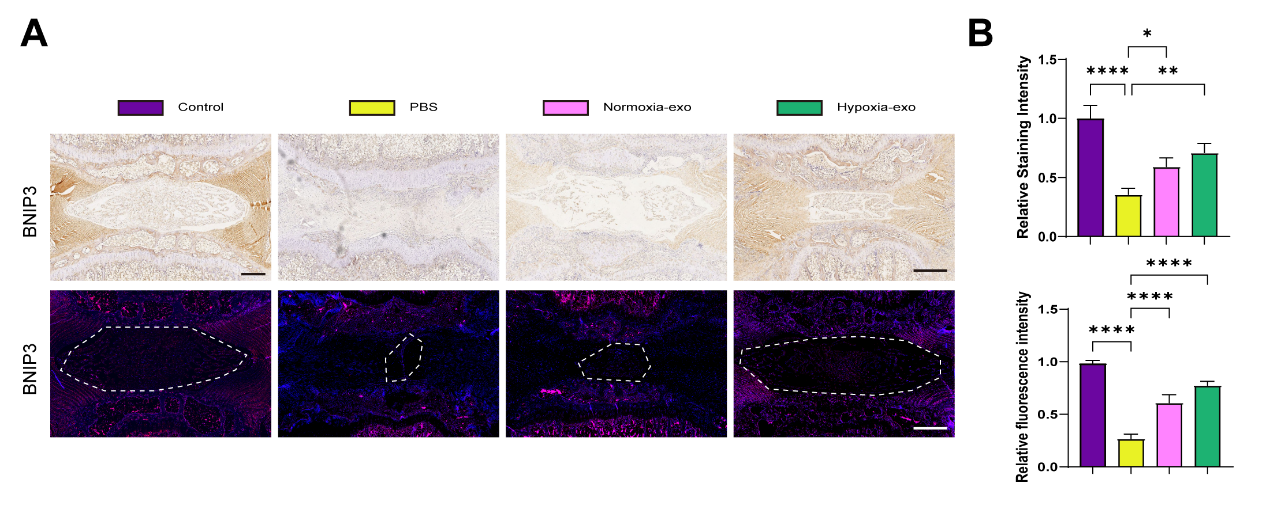


**Figure S3.** Following intervertebral disc degeneration (IVDD) surgery in rats, injections of PBS, Normoxia-exo, or Hypoxia-exo were administered. BNIP3 intensity was then evaluated using immunohistochemistry and tissue fluorescence techniques. n=4, Scale bar: 1mm. * *p* < 0.05; ** *p* < 0.01; **** *p* < 0.0001.


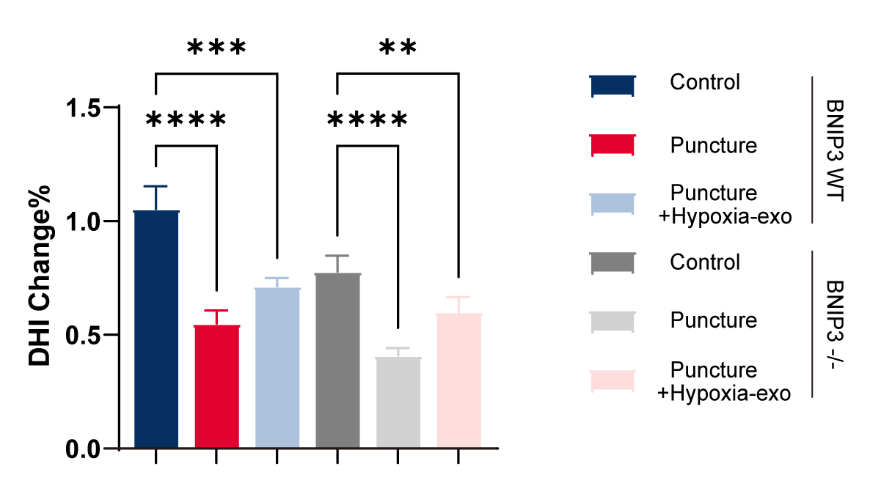


**Figure S4.** Quantitative analysis of DHI values in BNIP3 WT and BNIP3-/- mice following various treatments. n=4, ** *p* < 0.01; *** *p* < 0.001; **** *p* < 0.0001.


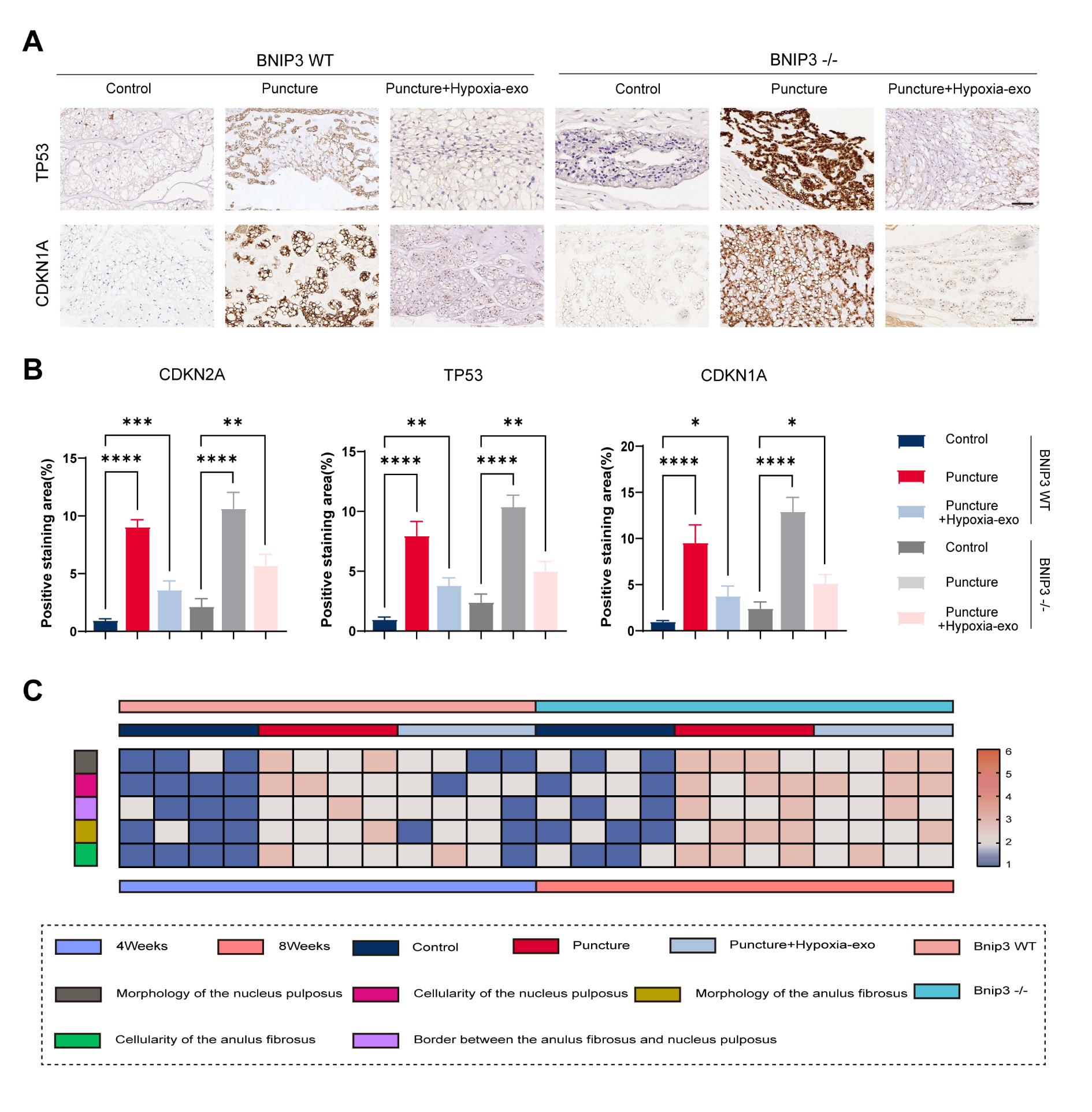


**Figure S5.** (A) Immunohistochemical analysis of TP53 and CDKN1A expression in BNIP3 WT and BNIP3 -/- mice. Scale bar: 200μm. (B) Quantitative immunohistochemical analysis of CDKN1A, CDKN2A, and TP53. n=4, * *p* < 0.05, ** *p* < 0.01, *** *p* < 0.001, **** *p* < 0.0001. (C) Using heatmaps to illustrate histological scores of HE and SO staining after different treatments in BNIP3 WT and BNIP3 -/- mice.

**
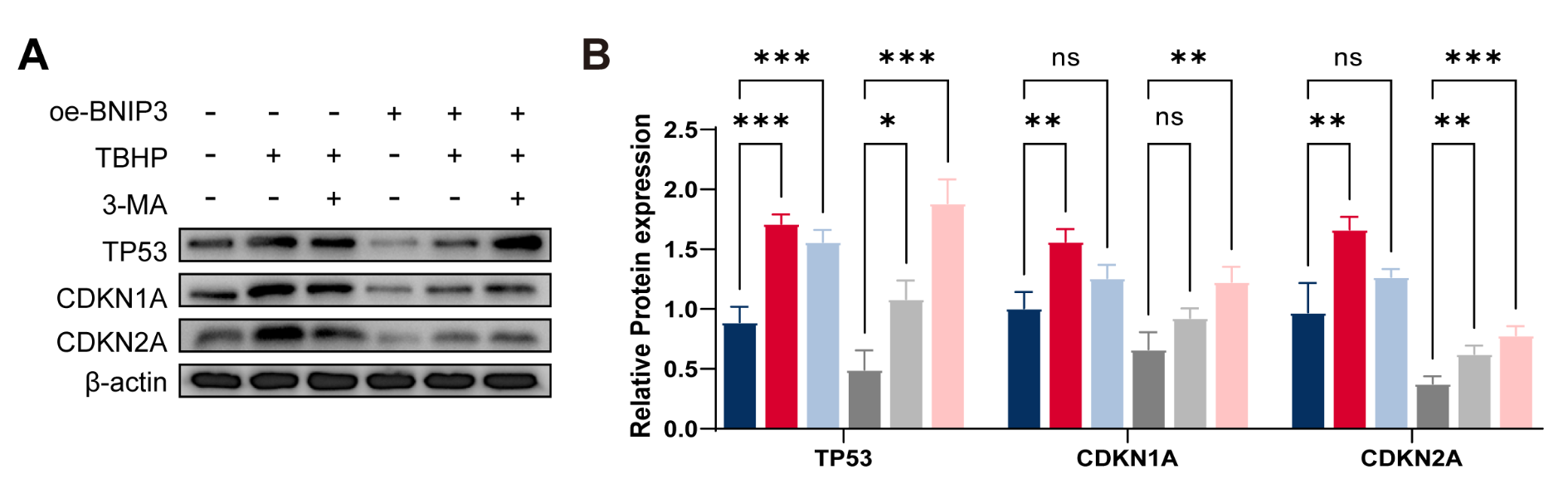
**

**Figure S6.** (A) Western-blot analysis was performed to assess the expression levels of senescence markers (TP53, CDKN1A, CDKN2A) in NP cells overexpressing BNIP3 following treatment with PBS, TBHP, or 3-MA. (B) Quantification of relative protein expression of TP53, CDKN1A, and CDKN2A. n=3, * *p* < 0.05, ** *p* < 0.01, *** *p* < 0.001, ns, not statistically significant. Data are expressed as mean ± SD.


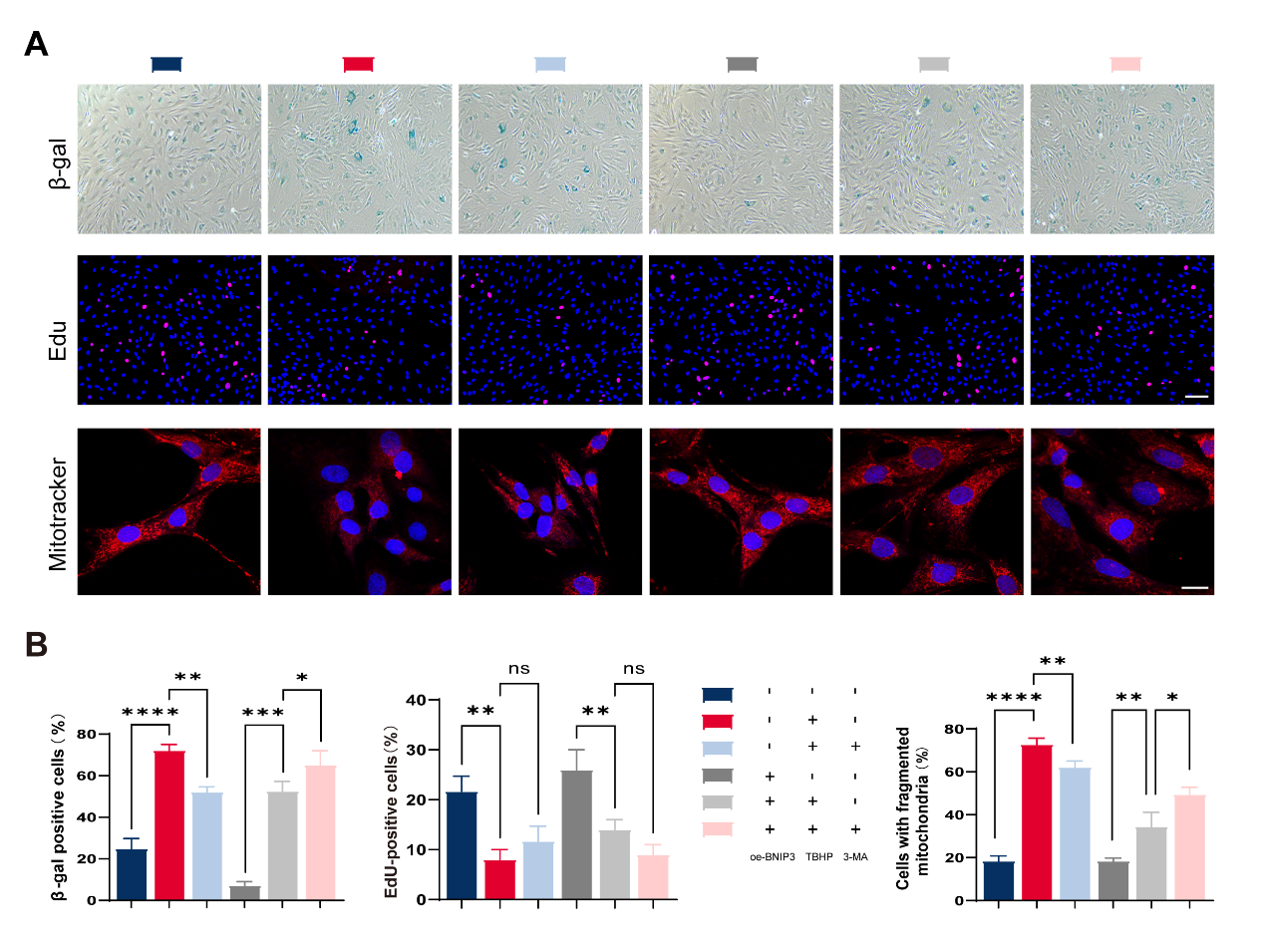


**Figure S7.** (A) NP cells were treated with PBS, TBHP, or 3-MA, respectively, and subsequently stained for cellular senescence (β-sal), Scale bars: 100 μm, NP cell proliferation (Edu), Scale bars, 100 μm, and mitochondrial morphology (Mitotracker) Scale bars, 20 μm, after BNIP3 overexpression. (B) Quantitative analysis of β-sal, Edu, and Mitotracker staining. n=3, * *p* < 0.05, ** *p* < 0.01, *** *p* < 0.001, **** *p* < 0.0001, ns, not statistically significant. Data are expressed as mean ± SD.

**
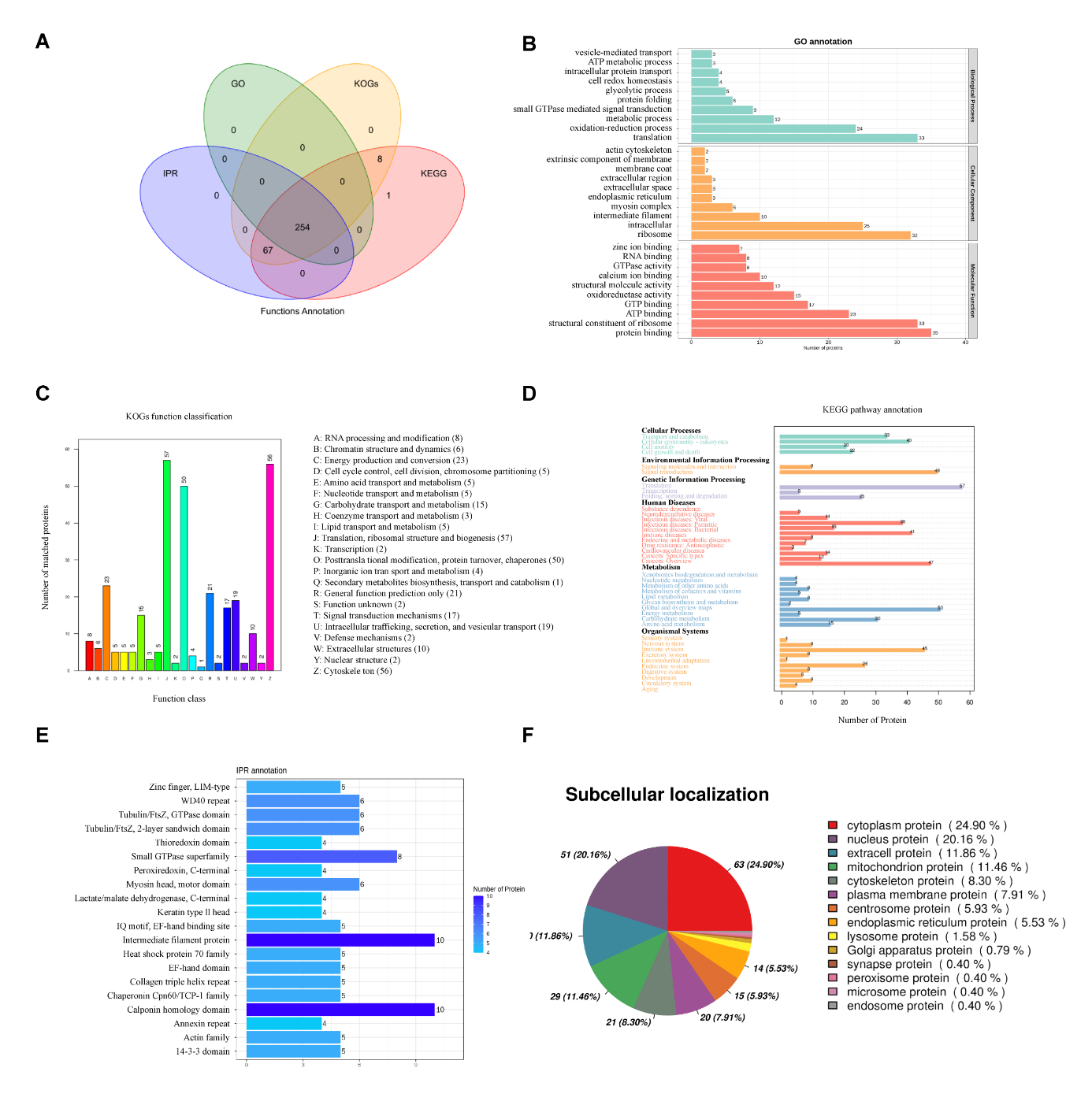
**

**Figure S8.** Graphical representation of BNIP3-interacting proteins. (A) Gene function annotations. (B) GO analysis. (C)KOGs functional enrichment classification. (D) KEGG functional analysis. (E) IPR functional annotation. (F) Subcellular localization analysis.


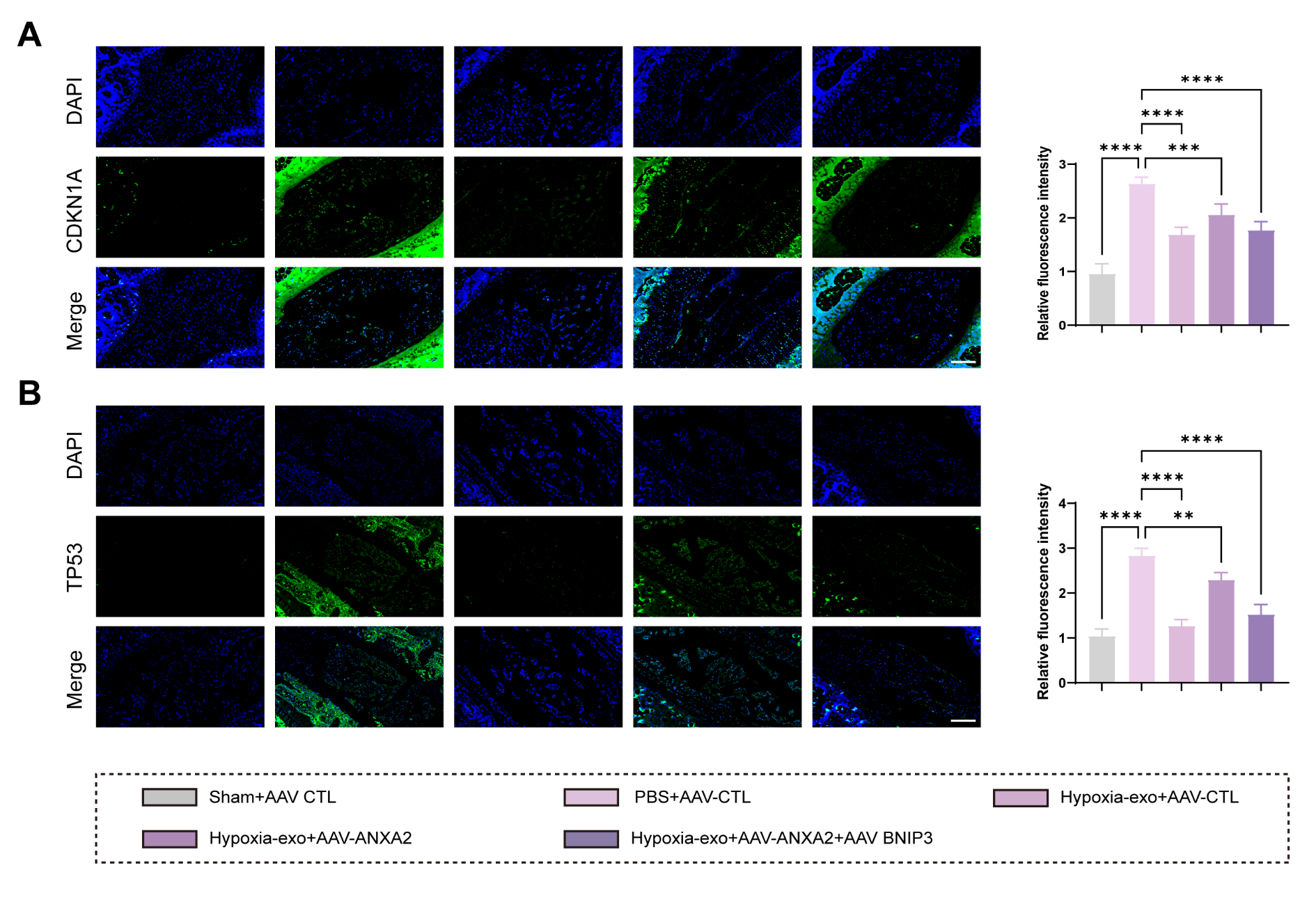


**Figure S9.** (A) Immunofluorescence staining and quantitative analysis of CDKN1A. n=4, Scale bar: 200μm. *** *p* < 0.001, **** *p* < 0.0001. (B) Immunofluorescence staining and quantitative analysis of TP53. n=4, Scale bar: 200μm. ** *p* < 0.01, **** *p* < 0.0001.


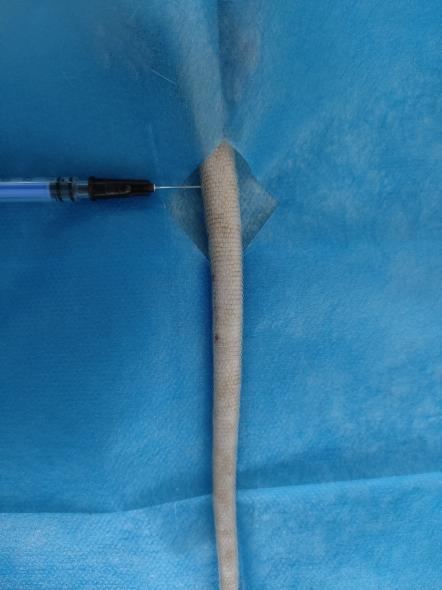

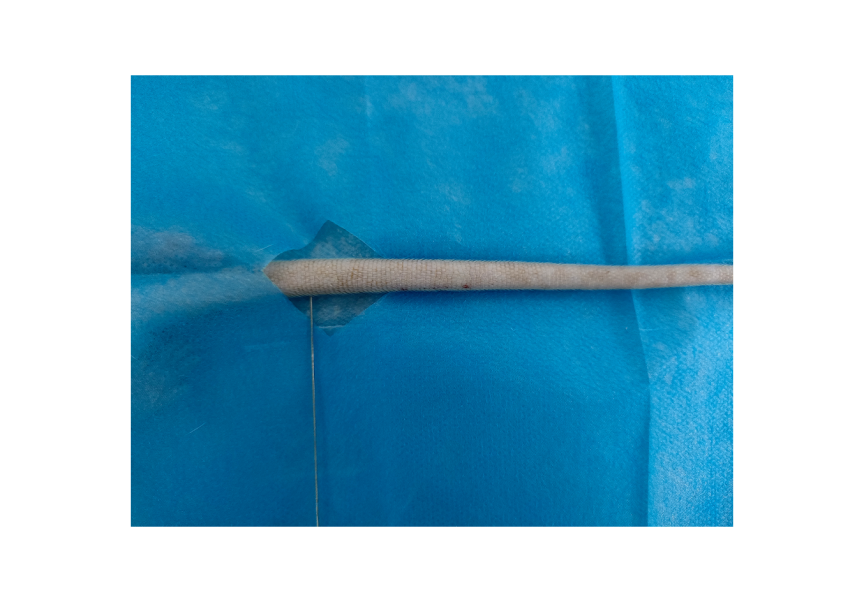


**Figure S10.** Intraoperative Procedures for IVDD and Exosome Injection.

(A) The left panel shows fine needle aspiration for the intravertebral disc degeneration (IVDD) procedure.

(B) The right panel illustrates a microinjector utilized for exosome injection.


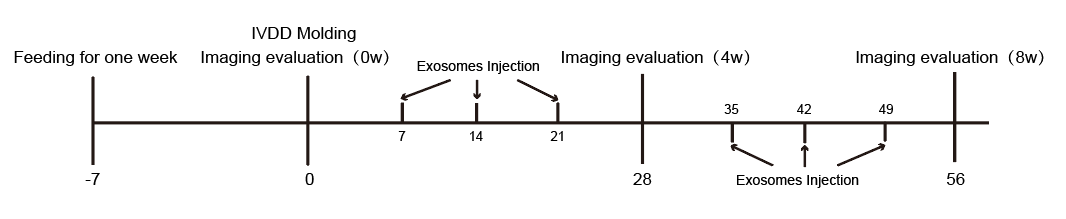


**Figure S11.** Experimental flowchart of animal models and exosome injection.

**SUPPLEMENTARY TABLES**

**Table S1.** Pfirrmann classification


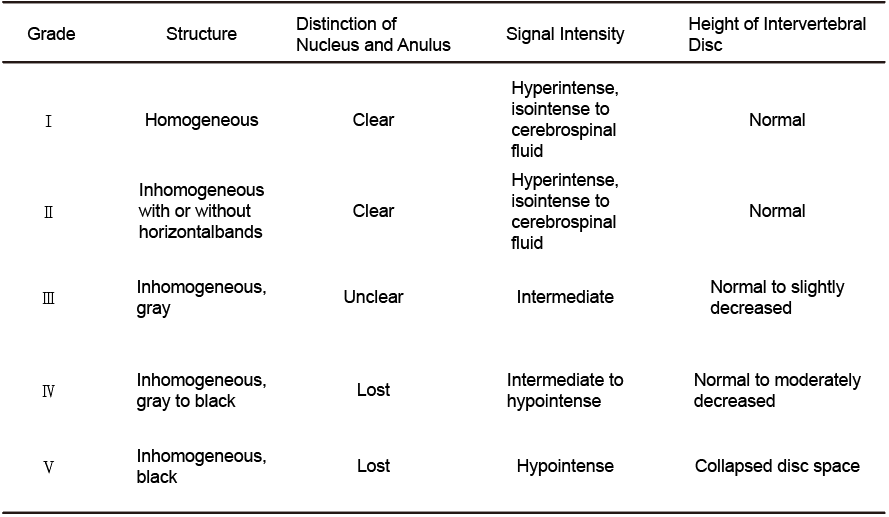


**Table S2.** Histological Grading Scale


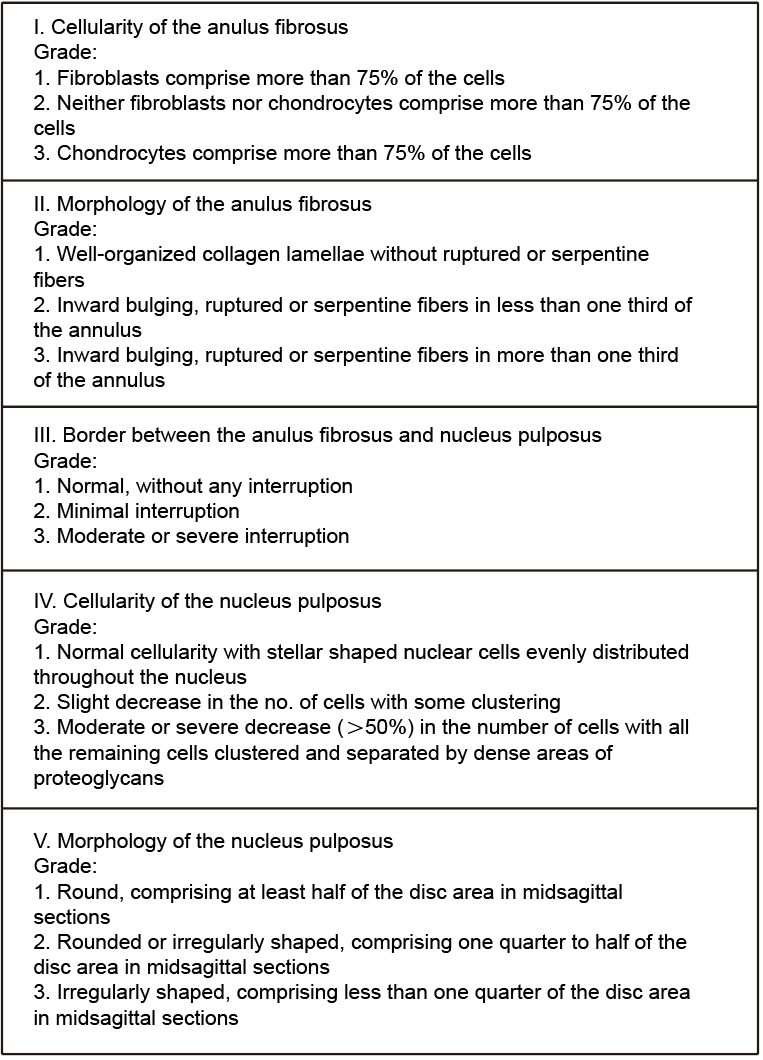


**Table S3.** The first ten proteins interacting with BNIP3 identified by LC-MS/MS.


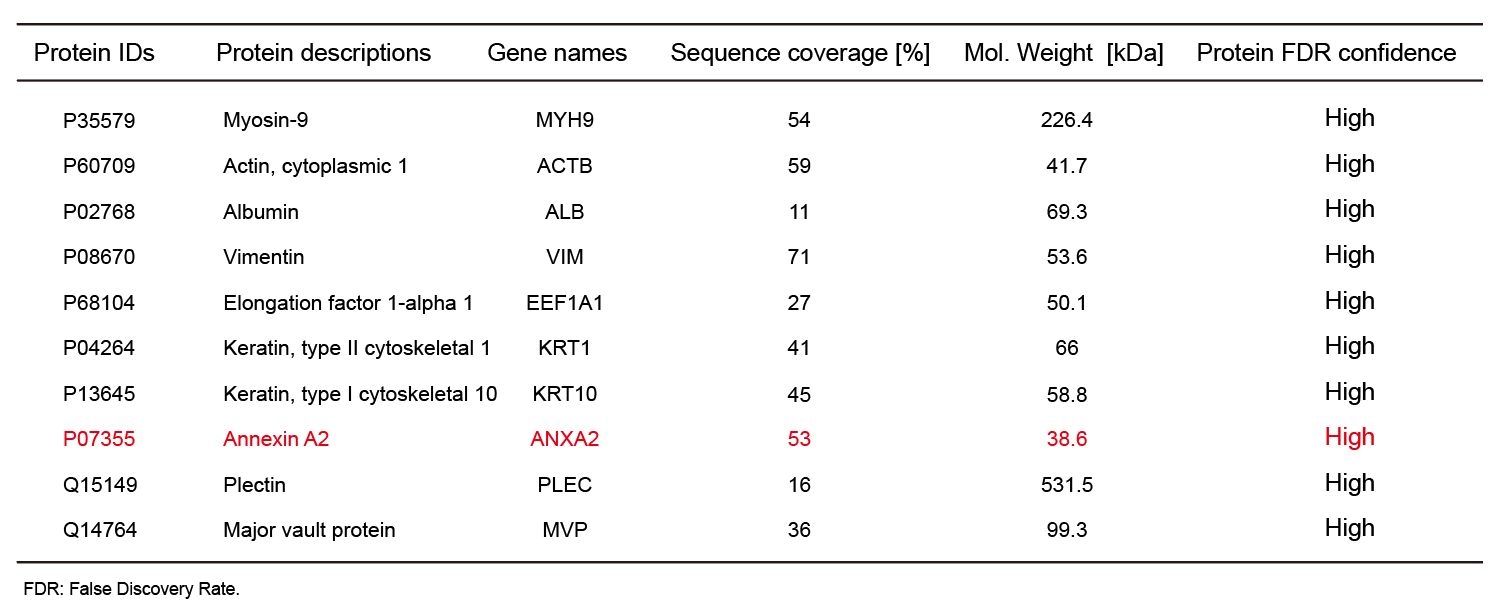


**Table S4.** Primary antibodies used in this study


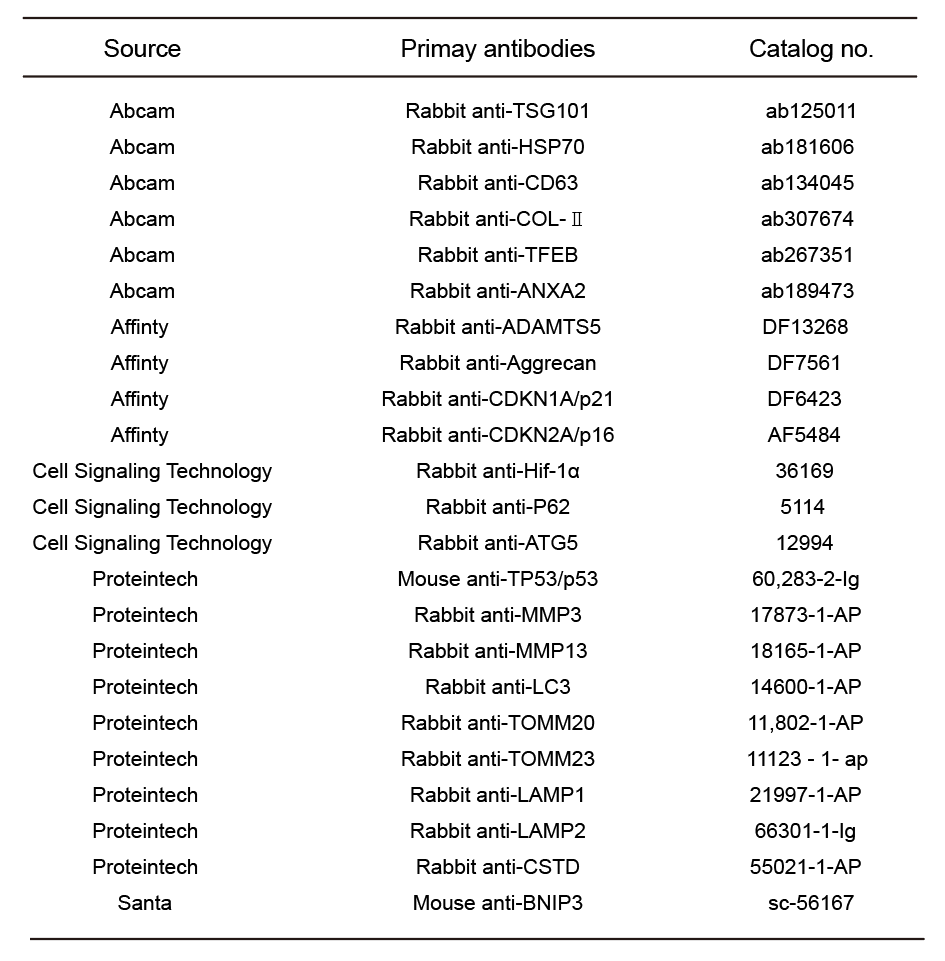


**Table S5.** Primers used for qPCR


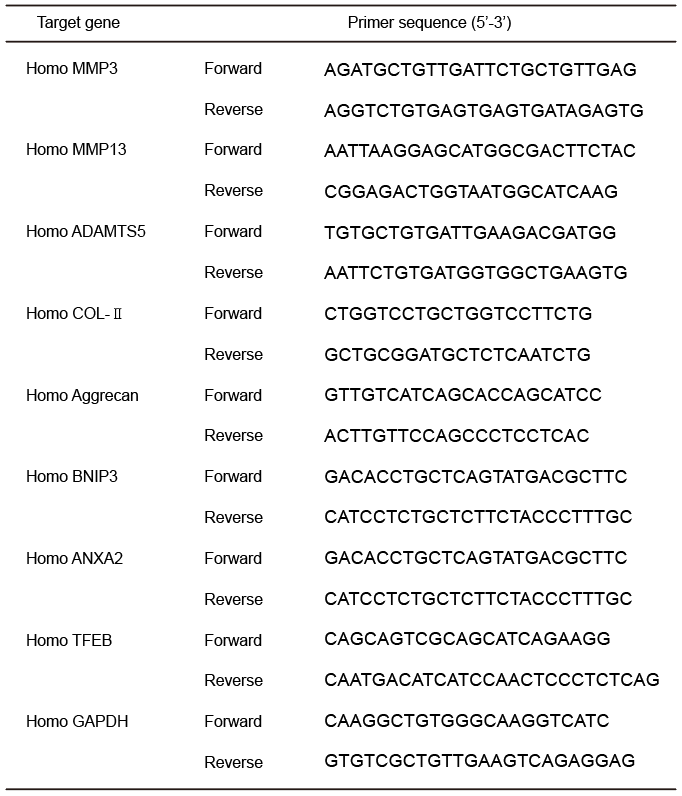


**Table S6.** siRNA sequence

**HIF1A**

| id | SS 5’-3’ | AS 5’-3’ |
| --- | --- | --- |
| 1576 | CGAUGGAAGCACUAGACAAAG | UUGUCUAGUGCUUCCAUCGGA |
| 1876 | CCAGCAGACUCAAAUACAAGA | UUGUAUUUGAGUCUGCUGGAA |
| 606 | GAAGAACUAUGAACAUAAAGU | UUUAUGUUCAUAGUUCUUCCU |

**BNIP3**

| 384 | CCAAGGAGUUCCUCUUUAAAC | UUAAAGAGGAACUCCUUGGGG |
| --- | --- | --- |
| 98 | GCCUCGGUUUCUAUUUAUAAU | UAUAAAUAGAAACCGAGGCUG |
| 286 | GGAAGAUGAUAUUGAAAGAAG | UCUUUCAAUAUCAUCUUCCUC |

**ANXA2 annexin A2**

|  |  | SS | AS |
| --- | --- | --- | --- |
| 1 | 909 | GGUCUGAAUUCAAGAGAAAGU | UUUCUCUUGAAUUCAGACCUA |
| 2 | 432 | GAGUCUACAAGGAAAUGUACA | UACAUUUCCUUGUAGACUCUG |
| 4 | 726 | GCAUCAGGAAAGAGGUUAAAG | UUAACCUCUUUCCUGAUGCUU |

**TFEB transcription factor EB**

|  |  | SS | AS |
| --- | --- | --- | --- |
| 1 | 748 | GAAAGACAAUCACAACUUAAU | UAAGUUGUGAUUGUCUUUCUU |
| 2 | 699 | GGCAGAAGAAAGACAAUCACA | UGAUUGUCUUUCUUCUGCCGC |
| 3 | 1137 | GGAGCAGCUUCAGCAUGGAGG | UCCAUGCUGAAGCUGCUCCGG |

The labeled sequences were selected for this study.
